# Pathogenicity and virulence of *Brucella*: Strategies for metabolic adaptation and immune evasion

**DOI:** 10.1080/21505594.2025.2585620

**Published:** 2025-11-10

**Authors:** Erika S. Guimarães, Marco Tulio R. Gomes, Ana Carolina V. S. C. de Araujo, Karla Karoline S. Ramos, Sergio C. Oliveira

**Affiliations:** a Departamento de Bioquímica E Imunologia, Instituto de Ciências Biológicas, Universidade Federal de Minas Gerais, Belo Horizonte, Brazil; b Departamento de Imunologia, Instituto de Ciências Biomédicas, Universidade de São Paulo, São Paulo, Brazil; c Institut Pasteur de São Paulo, São Paulo, Brazil

**Keywords:** *Brucella*, unfolded protein response, immune evasion, metabolic reprogramming, type I interferon, *Brucella* virulence factors

## Abstract

*Brucella* species are facultative intracellular pathogens that have evolved sophisticated mechanisms to evade host immune responses and establish chronic infections. This review provides an analysis of *Brucella* virulence and pathogenicity, with particular emphasis on the intricate interactions between the pathogen and its host. We examine the molecular and cellular mechanisms underlying *Brucella* pathogenesis, detailing the processes by which the bacterium invades, survives, and replicates within host cells. An in-depth discussion of the key *Brucella* virulence factors and their roles in immune evasion is provided, including strategies that enable the pathogen to circumvent immune detection, subvert host immune signaling pathways, and manipulate intracellular trafficking. Furthermore, we explore *Brucella* ability to modulate host cellular functions, particularly through the induction of the unfolded protein response and its implications for bacterial persistence. The review also highlights the influence of type I interferon responses on host susceptibility to *Brucella* infection, shedding light on their role in disease progression. Additionally, we discuss *Brucella* metabolic adaptations, including its ability to exploit host-derived nutrients and reprogram metabolic pathways to sustain long-term persistence. Finally, we address emerging research directions and future perspectives in the field, emphasizing the need for novel therapeutic and vaccine strategies. A deeper understanding of these complex host-pathogen interactions will be instrumental in developing more effective approaches for the control, prevention, and treatment of brucellosis.

## Introduction

Brucellosis is a highly prevalent yet often neglected bacterial zoonosis, causing an estimated annual economic loss of US$ 600 million in Latin America alone [1]. The disease is endemic in resource-limited regions such as the Middle East, Mediterranean, and Latin America [2]. *Brucella spp*, the causative agent of brucellosis, consists of Gram-negative facultative intracellular *Alphaproteobacteria*, that can persist and invade host cells, leading to chronic granulomatous infections [3,4]. Among the various *Brucella* species, *Brucella abortus* is the primary etiological agent of human brucellosis and is the most widely distributed globally [5]. This bacterium has a particular tropism for the reproductive organs of bovines, often resulting in spontaneous abortion in ruminants [6]. Zoonotic transmission of *B. abortus* typically occurs through the oral route via consumption of animal-derived products, such as unpasteurized dairy and raw meat from infected animals. However, infection can also occur through inhalation or subcutaneous pathways [7].

Recent reports estimate that brucellosis results in 1.6 to 2.1 million new human cases annually [8]. Although rarely fatal, human brucellosis is a chronic illness that can be disabling, affecting various organs and tissues with variable incidence [9]. Common symptoms include fever, fatigue, arthralgia, sweating, lymphadenopathy, hepatomegaly, and splenomegaly [10,11]. Moreover, *Brucella* infection can lead to a range of complications, such as undulant fever due to episodes of bacteremia, followed by the development of new infection foci, as well as arthritis, endocarditis, osteomyelitis, and neurological complications [12]. Patients undergoing antibiotic treatment often experience high relapse rates, and no vaccines are currently available for humans [13,14].

The immune evasion mechanisms and virulence factors of *Brucella* play a crucial role in the pathogenesis of brucellosis. This pathogen can proliferate and survive inside phagocytic cells, such as macrophages and dendritic cells (DCs), which function as replicative niches and vectors for systemic dissemination to other organs. Upon entering the host cell, *B. abortus* is enclosed within a double-membrane compartment called *Brucella*-containing vacuole (BCV) [15,16]. During intracellular establishment and proliferation, the BCV undergoes various molecular changes as it interacts with distinct host trafficking pathways [17,18]. Firstly, the BCV actively interacts with the endocytic pathway, acquiring early endosomal molecules (eBCV), which are later replaced by late endosomal markers. Meanwhile, controlled fusion with lysosomes leads to vacuole acidification, which kills most bacteria but is essential for the expression of the Type IV Secretion System (T4SS) VirB, a key *Brucella* virulence factor [16,18]. The T4SS modifies the primary secretory pathway, inducing the biogenesis of the replicative BCV (rBCV), via fusion of mature eBCVs with endoplasmic reticulum (ER) [18,19]. *Brucella* effectively replicates in the ER, and rBCVs are subsequently incorporated into autophagosome-like multi-membrane structures (aBCV), which facilitate bacterial egress and cell-to-cell spread within the host [20]. The ability of *Brucella* to grow and survive within professional phagocytes prompts its systemic spread and infection of other myeloid cells, such as liver and spleen macrophages, where *Brucella* can persist in granulomatous lesions [9].

## *Brucella* major virulence factors

Virulence factors are specialized molecules or structures produced by pathogens to enhance their ability to infect and persist within a host. *Brucella*, unlike many other pathogens, does not rely on classical virulence factors such as exotoxins, cytolysins, or exoenzymes. Instead, its pathogenicity is driven by unique factors, including LPS, the T4SS and the BvrR/BvrS system. This section explores these key virulence factors and their critical roles in *Brucell*a ability to infect, evade immunity, and cause a persistent infection.

### Lipopolysaccharide

*Brucella* strains have evolved unique mechanisms of virulence compared to other well-described bacteria that infect mammals. One example is the presence of an unusual smooth lipopolysaccharide (S-LPS) in the outer membrane of classical virulent *Brucella* species, such as *B. abortus*. The S-LPS comprises three main domains: a polysaccharide O-chain, a core, and lipid A [21]. Compared to other Gram-negative bacteria, *Brucella* LPS contains longer chains of lipid A, primarily consisting of C16, C18, C28, and other very long-chain fatty acids. As a result, *Brucella* LPS requires concentrations 100 times higher than those of Enterobacteriaceae LPS to kill the host [22]. Additionally, the core and O-chain of *Brucella* LPS play multiple roles in immune evasion: (1) protect against the bactericidal actions of complement proteins and antimicrobial peptides produced by host phagocytes, (2) interact with lipid rafts, facilitating entry into host cells via endocytosis and preventing extensive fusion of BCV with lysosomes, (3) inhibit antigen presentation by forming complexes with major histocompatibility complex class II (MHC-II), and (4) prevent caspase-2 mediated apoptosis in phagocytes [23–27]. Studies have identified several glycosyltransferases as essential for the biosynthesis and structural integrity of the LPS core. Among them, WadC, WadB, and WadD function as key enzymes that incorporate specific sugar residues into the LPS core region [28,29]. In particular, mutations in *wadC* prevent the incorporation of the mannose residue that anchors the lateral branch, leading to significant consequences: *wadC* mutants display increased sensitivity to host antimicrobial defenses, impaired intracellular replication, enhanced recognition by the CD14-MD2-TLR4 receptor complex, heightened production of inflammatory cytokines, and accelerated dendritic cell maturation [28,30]. WadB also contributes to LPS core assembly, and its disruption alters LPS composition and surface properties, resulting in increased sensitivity to cationic antimicrobial peptides and reduced persistence in host cells [31]. Likewise, WadD has been characterized as a novel LPS core glycosyltransferase, and its inactivation disrupts LPS structure, reducing resistance to polycationic peptides [29].

### Type IV secretion system

The VirB T4SS apparatus, is encoded by the *virB1*- *virB12* genes on Chromosome II. Initially identified in *B. suis*, the *virB* operon has since been found to be highly conserved across all *Brucella* species [32]. Although the precise molecular mechanisms initiating T4SS assembly remain unclear, intracellular signals are thought to play a crucial role in inducing its assembly. One such signal is the acidic pH environment within the BCV, resulting from lysosomal fusion and acidification, which likely triggers the upregulation of the *virB* operon [33]. Several regulatory factors, including the LuxR-type regulator VjbR [34], the integration host factor (IHF) [35], the *Brucella* LuxR-like regulator (BlxR) [36], and the *Brucella* quorum-sensing regulator (BabR) [37], modulate the expression of the *virB* operon. However, their precise roles in T4SS induction during infection are not fully understood. A functional T4SS is essential for *Brucella* survival and persistence within host cells as mutants lacking any VirB genes are unable to replicate intracellularly [38,39]. One mechanism by which the T4SS promotes intracellular survival is by regulating the trafficking of BCV within macrophages, ensuring that the bacteria avoid degradation in phagolysosomes [19,40]. *Brucella* replicates exclusively within the ER after the formation of rBCVs. The acquisition of ER membranes, which is crucial for establishing a replication niche, is dependent on a functional T4SS system [40]. In contrast, *virB* mutants are persistently trafficked to lysosomes, leading to bacterial degradation. T4SS effectors interact with ER exit sites (ERES) to establish replication niches, preventing lysosomal fusion. Interestingly, the VirB apparatus appear to be more important for late BCV maturation stages than for early survival events [41]. Furthermore, a functional T4SS is necessary for *B. melitensis* and *B. abortus* to elicit a robust innate immune response during late stages of infection in mice [42]. *virB* mutants induce lower IFN-γ production by CD4 T cells, resulting in a diminished Th1-polarized immune response [43]. Notably, it remains unclear whether these immune effects arise directly from T4SS activity or indirectly from lysosomal degradation of *virB*-deficient mutants. Moreover, Li *et al* [44]., demonstrated that recombinant T4SS effectors can stimulate both Th1 and Th2-associated cytokines in macrophages, highlighting their direct immunomodulatory activity. More recently, RS15060 was identified as a novel T4SS effector required for *Brucella* virulence, thereby expanding the known repertoire of bacterial factors that subvert host cellular pathways [45]. Another important effector, NpeA, is actively translocated into host cells and plays a critical role in intracellular replication. Notably, NpeA harbors a conserved short linear motif that binds directly to N-WASP, a central regulator of Arp2/3-dependent actin nucleation. This interaction promotes cytoskeletal remodeling, which facilitates the maturation of the BCV into a replication-permissive niche [46].

### VceA and VceC

VirB-co-regulated effector (Vce) A and VceC are two effector proteins translocated into macrophages via the VirB T4SS [47]. The deletion of the VceA effector enhances autophagy in infected human trophoblast cells, suggesting that VceA plays a crucial role in suppressing autophagy to facilitate intracellular survival during infection [48]. Additionally, VceA-deleted mutants stimulate the secretion of tumor necrosis factor-alpha (TNF-α) and interleukin (IL)-1β, which are involved in inhibiting apoptosis, likely as a strategy to preserve the host cell and maintain the intracellular niche [48]. VceC is a conserved effector that translocates to the ER and interacts with the ER chaperone GRP78/BiP, triggering ER stress. This interaction activates the unfolded protein response (UPR), ultimately leading to an inflammatory response through the inositol-requiring enzyme-1-alpha (IRE1α) pathway [47,49]. In addition to its role in ER stress, VceC regulates apoptosis in a cell type-dependent manner. In placental trophoblasts, VceC promotes apoptosis, potentially dampening immune responses and facilitating bacterial escape [50]. Conversely, in goat trophoblast cells, VceC inhibits apoptosis, preserving the intracellular niche and promoting *Brucella* proliferation [51].

### TcpB/BtpA and BtpB

The T4SS effectors Toll/Interleukin 1 like receptor domain containing protein (TcpB/BtpA) and BtpB contain a Toll-interleukin-1 receptor (TIR) domain, which structurally mimics those found in host innate immune receptors, allowing *Brucella* to subvert the host immune response [52–55]. Although TcpB and BtpB target similar pathways [56], the role of BtpB in modulating *Brucella*-induced inflammatory responses and bacterial persistence remains unclear. Recent studies have demonstrated that deletion of BtpB increases microtubule-associated protein 1 light chain 3B (LC3-II) expression, decreases p62 levels, and leads to the accumulation of autophagic lysosomes, suggesting that BtpB participates in autophagy inhibition [57]. Beyond Toll-like receptor (TLR) signaling suppression, TcpB interacts with phosphatidylinositol-4,5-bisphosphate (PI(4,5)P2) or PI(3,4,5)P3. This interaction suppresses the activation of antigen-presenting cells, impeding CD8 T cells from targeting infected cells and further weakening host immune defenses [58]. Additionally, TcpB modulates microtubule dynamics by acting as a stabilizing factor [59]. This modulation influences *B. abortus* intracellular survival, the maturation of pathogen-containing vacuoles, and the production of pro-inflammatory cytokines in infected macrophages [60]. TcpB has also been shown to induce the UPR in infected macrophages, although the exact mechanisms underlying this effect remain undefined [61]. Moreover, TcpB and BtpB exhibit NAD^+^ hydrolase activity, enabling *Brucella* to deplete NAD^+^ levels in host cells during infection. This enzymatic activity leads to actin depolymerization, endocytic block, and a reduction in kinase activity [62].

### Two-component system BvrR/Bvrs

The two-component system BvrR/BvrS is a master gene regulator conserved across the *Alphaproteobacteria* class. BvrS is a sensor histidine kinase protein located in the inner membrane, while BvrR is a cytoplasmic response regulator [63]. BvrRS transcriptionally regulates numerous genes implicated in *B. abortus* virulence, carbon and nitrogen metabolism, and cell envelope homeostasis. As *Brucella* migrates to an intracellular lifestyle, its finely tuned gene expression enables adaptation to the stresses of the intracellular environment [64]. In this context, BvrRS is induced under acidic nutrient-limited conditions, helping *Brucella* bypassing host immune mechanisms during its initial interactions with eukaryotic cells [65]. An example of this regulatory system is the BvrRS/VjbR/VirB circuit [65]. During the early intracellular cycle of *B. abortus*, BvrS detects pH changes and nutrient starvation, leading to autophosphorylation. The phosphate group is transferred to BvrR (BvrR-P), which binds to target promoters, including one that regulates the expression of the transcriptional factor VjbR. Both VjbR and BvrR subsequently activate the *virB* operon, driving the expression of genes encoding the T4SS [65]. Moreover, the BvrRS/VjbR/VirB circuit plays a role in sensing and coordinating bacterial release and interactions with new target host cells during the late stages of infection, when *B. abortus* migrates from ER to the aBCV [66]. Regarding cell envelope regulation, BvrRS controls the expression of genes encoding for outer membrane proteins (Omps), such as Omp25, as well as genes involved in lipid A acylation [67]. *B. abortus* mutants in *bvrR* and *bvrS* are avirulent, exhibiting reduced invasiveness and an inability to replicate in cells and a mouse model [63].

### Outer membrane proteins

On the cellular surface of *Brucella*, the outer membrane proteins (Omp25/Omp31) and lipoproteins (Omp10/Omp16 and Omp19) further contribute to bacterial pathogenicity [68]. Omp16 is a conserved peptidoglycan-associated lipoprotein that plays a crucial role in bacterial virulence. This protein contributes to the structural integrity of the bacterial cell, enhancing resistance to environmental stresses. Deletion or downregulation of the *omp16* gene significantly reduces the bacterium’s ability to survive and replicate intracellularly, highlighting its importance in maintaining infection [69,70]. Omp19, the best-characterized lipoprotein from *Brucella*, presents strong immunomodulatory effects. Omp19 inhibits interferon-γ (IFN-γ)-induced MHC-II expression in macrophages during *B. abortus* infection by binding to the TLR2 receptor for prolonged periods and disrupts antigen processing and presentation by macrophages to T CD4 lymphocytes, significantly impairing the adaptive immune response in patients with brucellosis [71,72]. Evidence also indicates that Omp19 has notable protease inhibitor activity, avoiding lysosome proteases in host macrophages and intestinal proteases during oral infections [73]. The importance of these molecules as virulence factors is highlighted by the attenuation of *B. abortus* Omp19 and Omp25 mutants in mice and natural hosts [68,74]. Given the pivotal role of these Omps in host-pathogen interactions, *Brucella* must ensure their proper folding and insertion into the outer membrane to maintain virulence. These essential processes are facilitated by conserved molecular systems, such as the β-barrel assembly machinery (BAM) complex. The *Brucella* genome encodes homologs of BamA, BamD, and BamE, but lacks BamB and BamC, which are present in *Escherichia coli* and other *Gammaproteobacteria* [75,76]. Complementing BAM complex’s function, EipB is a conserved periplasmic protein essential for maintaining outer membrane integrity. Although not directly linked with individual Omps, mutants deficient in EipB exhibit increased outer membrane fragility, heightened sensitivity to environmental stress, and significant attenuation in vivo. Structural and genetic evidence further suggests that EipB may act synergistically with the BAM complex to support Omp biogenesis [77].

### Cyclic β-1,2-glucans

Cyclic β-1,2-glucans (CbG) are ring-shaped molecules consisting of 17–25 glucose units linked by β‐1,2-glycosidic bonds [78]. In many bacterial species, CbG production is regulated by osmotic conditions, highlighting its role in osmoprotection [79]. However, in *Brucella*, CbG synthesis is not osmoregulated [80], and experimental evidence suggests that this polysaccharide plays a minimal role in *Brucella* osmoprotection [81]. Nevertheless, CbG is critical for *Brucella* intracellular survival. *Brucella* mutants that cannot synthesize or transport CbG to the periplasmic space exhibit reduced virulence [81–83]. Recent structural and functional analyses have underscored the importance of CbG export via the Cgt ABC transporter, as Δcgt mutants retain CbG intracellularly but fail to deliver it to the periplasm, resulting in defective intracellular replication and significant attenuation in murine infection models [84]. CbG interacts with lipid rafts on host cell membranes, disrupting their organization and preventing phagosome-lysosome fusion. This interference allows *Brucella* to evade degradation and replicate within the ER [82]. Furthermore, CbG modulates the production of both proinflammatory and anti-inflammatory cytokines in macrophages and DCs [81,85,86]. In murine models, *Brucella* CbG has been linked to spleen inflammation due to the recruitment of monocytes, DCs and neutrophils, driven by the induction of cytokines, such as IL‐12 and TNF‐α [81]. Additionally, CbG acts as a potent activator of DCs, triggering antigen-specific CD8 T cell responses in vivo and enhancing antigen-specific CD4 and CD8 T cell responses [86].

### BspA, BspB, BspF, BspG, BspJ and BspL

BspA inhibits the host ER-associated degradation (ERAD) pathway by targeting the E3 ubiquitin ligase membrane-associated RING-CH-type finger 6 (MARCH6). This inhibition disrupts the degradation of ERAD substrates, facilitating the intracellular proliferation of *B. abortus* [87]. Moreover, BspA, along with other effectors like BspB and BspF, contribute to the inhibition of host protein secretion. This combined interference allows *Brucella* to modulate host secretory pathways, creating a favorable environment for its replication [88]. BspB specifically targets the conserved oligomeric Golgi (COG) tethering complex, altering Golgi membrane trafficking and redirecting Golgi-derived vesicles to the BCV. This process is vital for the biogenesis of rBCVs and optimal replication within host cells [89]. Additionally, BspF regulates host cell apoptosis by attenuating the crotonylation modification of the tumor suppressor protein p53, leading to reduced p53 expression. This suppression in turn inhibits the transcription of downstream apoptotic genes, thus preventing apoptosis [90]. BspF also promotes *Brucella* replication within rBCVs by interfering with vesicular transport between the trans-Golgi network and the plasma membrane. This interference occurs through modulation of the Arf6-Rab8a GTPase cascade, a key player in vesicular trafficking [91]. Furthermore, BspG interacts with host proteins involved in mitochondrial respiratory pathways to promote anti-apoptotic mechanisms and enhance the intracellular survival of *B. abortus* [92]. BspJ has been identified as a nucleomodulin that plays a pivotal role in regulating host energy synthesis, metabolism, and the inhibition of apoptosis signaling pathways. These functions collectively support the intracellular survival of *Brucella*. BspJ deletion significantly impairs the survival and proliferation of *B. abortus* during the rBCV phase and affects the secretion of inflammatory factors in both host cells and mice [93,94]. BspL has been characterized as a *Brucella* effector that specifically targets the host ERAD machinery. By enhancing ERAD activity during the late stages of infection, BspL delays the formation of aBCVs and prevents premature bacterial release from host cells, likely ensuring optimal intracellular replication and promoting efficient cell-to-cell dissemination [95].

### RicA

Rab2-interacting conserved protein A (RicA) targets host cellular pathways by specifically interacting with Rab2, a small GTPase involved in vesicular trafficking between the ER and the Golgi apparatus, which is crucial for *B. abortus* intracellular infection [96]. By hijacking Rab2-mediated trafficking, RicA alters the normal kinetics of BCV maturation, preventing its fusion with lysosomes [40,97]. This interference allows *Brucella* to avoid lysosomal degradation and supports its establishment within a replicative niche. Mutants lacking RicA exhibit a decrease recruitment of Rab2 to BCVs and significant defects in BCV maturation, underscoring its involvement in modulating host vesicular trafficking [40,96]. However, RicA-deficient mutants display no significant reduction in virulence in infected mice or HeLa cells [98]. Surprisingly, RicA mutants lose the late endosomal marker LAMP1 earlier than wild-type bacteria, indicating accelerated escape from late lysosomes. This earlier arrival at the ER allows RicA mutants to establish their replicative niche faster than wild-type *Brucella* [97]. This phenomenon appears to be further modulated by the effector BspB, which attenuates the negative impact of RicA on Rab2 function [99]. The interplay between RicA and BspB highlights the intricate regulatory network employed by *Brucella* to fine-tune host vesicular trafficking for optimal infection outcomes.

### Antioxidant activity

*Brucella spp*. exhibit remarkable resistance to the bactericidal activity of professional phagocytes, allowing them to persist in high numbers within neutrophils and evade oxidative killing mechanisms [100]. The antimicrobial activity of these cells relies heavily on the generation of reactive oxygen species (ROS), such as superoxide anion (O_2_^−^) and hydrogen peroxide (H_2_O_2_). Given that H_2_O_2_ can freely diffuse across biological membranes, *Brucella* detoxifies it using both the periplasmic catalase KatE and the cytoplasmic peroxiredoxin AhpC, which work together to neutralize intracellular H_2_O_2_ [101,102]. Notably, either KatE or AhpC alone is sufficient to maintain chronic infection in murine models. However, a *B. abortus* double mutant lacking both enzymes shows significant attenuation in IFN-γ-activated macrophages, underlining their complementary roles [102]. Beyond H_2_O_2_ detoxification, *Brucella* also employs superoxide dismutases (SODs) to protect against superoxide radicals generated by both host cells and its own metabolism. Cu/Zn-cofactored SOD (SodC) is localized in the periplasm and primarily counters extracellular superoxide. The SodC mutant shows increased susceptibility to macrophage killing following IFN-g stimulation, a phenotype partially reversed by inhibition of NADPH oxidase using apocynin, supporting its role in defending against the respiratory burst [103]. In contrast, Mn-cofactored SOD (SodA) is cytoplasmic and targets superoxide species generated endogenously by the bacterial metabolism, which are less likely to cross membranes due to their charge. Disruption of sodA similarly impairs *B. abortus* survival in macrophages and during the early stages of infection in mice [104], further emphasizing the importance of these complementary antioxidant systems. Recent studies have revealed that *B. melitensis* also manipulates host cell ferroptosis pathways to enhance intracellular survival and promote dissemination. Specifically, during the early phase of infection, *Brucella* activates the GTP cyclohydrolase 1 (GCH1)-tetrahydrobiopterin (BH_4_) axis, a GPX4-independent antioxidant mechanism that suppresses lipid peroxidation and ferroptotic cell death in macrophages, thereby creating a permissive niche for bacterial replication. In contrast, during the late phase of infection, the pathogen inhibits the classical GPX4-glutathione (GSH) axis, facilitating ferroptosis and allowing bacterial egress from host cells. This temporal regulation illustrates a sophisticated immune evasion strategy in which *Brucella* balances intracellular persistence with intercellular spread [105]. OxyR is a central transcriptional regulator that coordinates *Brucella*’s antioxidant defenses during infection. Deletion of *oxyR* impairs bacterial growth and reduces tolerance to multiple stressors, including oxidative agents, acidic conditions, and antimicrobial peptides, underscoring its role in stress adaptation. Loss of OxyR also downregulates key antioxidant enzymes such as peroxidases, catalase, and superoxide dismutase, indicating that OxyR orchestrates a coordinated antioxidant response. Notably, intracellular survival within macrophages remains largely unaffected, suggesting that OxyR primarily protects *Brucella* from environmental stress rather than hot-mediated intracellular killing. Additionally, *oxyR* deletion suppresses inflammatory cytokines expression, indicating a potential role in modulating host immune responses [106].

### Small regulatory RNAs

Small regulatory RNAs (sRNAs) have emerged as central players in bacterial adaptation and pathogenesis. These non-coding RNAs, typically 50 to 300 nucleotides long, modulate gene expression post-transcriptionally through base pairing with target mRNAs, thereby influencing mRNA stability and translation [107]. Many sRNAs function in concert with the RNA chaperone Hfq, which facilitates RNA-RNA interactions and stabilizes both sRNAs and their mRNA targets In *Brucella*, Hfq is essential for full virulence, and its deletion leads to pleiotropic effects, including enhanced sensitivity to oxidative stress, reduced intracellular survival, and downregulation of key virulence determinants such as the quorum sensing regulator BabR and the VirB system [37]. Among the best-characterized sRNAs in *B. abortus* are AbcR1 and AbcR2. These trans-encoded sRNAs exhibit partial sequence homology and regulate overlapping but distinct sets of targets, primarily mRNAs encoding amino acid and polyamine ABC transporters. Functional studies show that a double mutant lacking both *abcR1* and *abcR2* is significantly attenuated in murine models and exhibits reduced intracellular replication, whereas single deletions have minimal impact on virulence [108,109]. Their regulatory activity relies on two conserved seed sequences (M1 and M2), with M2 being essential for in vivo virulence. Mutation of this motif leads to dysregulation of key targets and impairs splenic colonization [109]. High-throughput RNA sequencing has expanded the known repertoire of sRNAs in *Brucella*, predicting numerous candidates, several of which have been experimentally validated, including BSR0602, BSR0441, BASI74, and Bmsr1 [110,111]. BSR0602 is strongly upregulated during stress and infection and represses the transcriptional regulator *gntR* via Hfq-dependent pairing, compromising intracellular survival and reducing bacterial burden in the spleen, suggesting a role in negatively regulating virulence [112]. In contrast, Bmsr1 appears to enhance pathogenicity: its expression is induced during macrophage infection, and deletion of *bmsr1* leads diminishes survival and splenic colonization. Transcriptomic profiling of the bmsr1 mutant revealed downregulation of key virulence genes, including *virB2*–*virB11* and VjbR, indicating that Bmsr1 modulates virulence programs in response to intracellular cues [113]. A more recently described sRNA, MavR (MurF- and virulence-regulating RNA), is essential for persistent infection in mice. MavR specifically regulates murF, which encodes an enzyme involved in the cytoplasmic steps of peptidoglycan biosynthesis, through a six-nucleotide seed motif. Disruption of this interaction leads to dysregulated *murF* expression, impaired replication, and reduced colonization during chronic infection, highlining MavR as a bona fide virulence-associated sRNA [114].

### Quorum sensing systems (QS)

QS is key regulatory mechanism that allows bacteria to coordinate gene expression in response to population density and environmental cues [115]. In *Brucella*, QS plays a significant, albeit non-canonical, role in modulating virulence. A hallmark of this system is the detection of N-dodecanoyl homoserine lactone (C_12_-HSL), a prototypical QS molecule, in the supernatant of *B. melitensis* cultures. Exogenous C_12_-HSL represses the expression of critical virulence genes, including components of the T4SS [116]. At the core of QS regulation are the LuxR-type regulators VjbR and BabR (also known as BlxR). VjbR is indispensable for full virulence as *vjbR*-deficient strains exhibit marked attenuation in both cellular and murine infection models [117,118]. BabR modulates a subset of VjbR-regulated genes, exerting both synergistic and antagonistic effects, though its deletion alone does not significantly impair virulence [36,119]. Recent evidence indicates that VjbR and BabR act cooperatively during chronic infection, with BabR autoregulating its own expression while VjbR mildly represses BabR, highlighting a complex regulatory interplay [120]. Further layers of regulation involve MucR, which directly represses *babR* expression, indirectly facilitating upregulation of VirB. This dual-layered control underscores the finely tuned regulatory network that governs virulence gene expression [121]. QS is also integrated with environmental sensing systems: the two-component system BvrR/BvrS activates *vjbR* during early host cell invasion [65], while transcriptional regulators GntR10 and GntR17 positively regulate both *vjbR* and *babR*, influencing the production of T4SS effectors such as BspE and BspF [122,123]. Despite these advances, several knowledge gaps remain. The endogenous biosynthetic pathway for C_12_-HSL in *Brucella* is still unknown, and while a lactonase capable of degrading C_12_-HSL has been characterized [124], upstream metabolic processes remain elusive. Additionally, VjbR function at certain promoters appears to require co-regulators that have yet to be fully defined [125].

### Moonlighting metabolic enzymes

*B. abortus* repurposes both bacterial and hots metabolic enzymes to enhance intracellular survival and modulate host-pathogen interactions. One prominent example involves the interaction between the T4SS effector BPE123 and host α-enolase (ENO-1). Marchesi *et al* [126]., demonstrated that ENO-1 localizes to BCVs in a BPE123-dependent manner, knockdown of ENO-1 in HeLa cells significantly impairs intracellular replication. In parallel, bacterial enolase and elongation factor Tu (EF-Tu) have been detected on the *B. abortus* surface during biofilm formation, suggesting additional roles in adhesion, immune interaction, or biofilm maintenance [127]. Biochemical studies further show that *B. abortus* enolase retains its canonical glycolytic activity while also binding host fibronectin and eliciting recognition by sera from infected cattle [128]. Another example of moonlighting behavior is the chaperonin GroEL, a highly conserved protein primarily involved in folding nascent and stress-denatured proteins. Beyond this canonical role, GroEL is surface exposed during infection and interacts directly with host immune components, functioning as an immunodominant antigen. This extracellular localization allows GroEL to influence host immune responses, potentially promoting bacterial persistence and immune evasion [129].

## *Brucella* evades host innate immune activation and signaling mechanisms

The innate immune system relies on its capacity to quickly identify invading pathogenic microbes as foreign and initiate actions to neutralize the threat [130]. This defense mechanism depends on the coordinated interaction between immune cells and invading pathogens to initiate an effective immune response [131]. However, *Brucella* has developed sophisticated strategies to evade host innate immune receptors, facilitating its survival and replication within host cells [132].

One of the first identified members of pathogen recognition receptors (PRRs), the TLR4 receptor, plays an important role in the detection of bacterial LPS [133]. LPS, an integral component of the outer membrane of Gram-negative bacteria, is a powerful immune stimulant and a major contributor to the onset of septic shock [134]. The identification of TLR4 as the receptor for LPS marked a significant breakthrough in understanding how the innate immune system recognizes and responds to microbial infections. However, several characteristics of *Brucella* LPS contribute to its ability to evade detection by the innate immune system. Notably, the numerous attenuated mutants with structural defects in their LPS underscore the critical role of this molecule in *Brucella* virulence [135]. A vital adaptation involves the modification of the lipid A moiety, which enables *Brucella* to avoid recognition by TLR4. Unlike the lipid A of many bacterial pathogens that typically contains short-chain fatty acids (C12-C16), *Brucella* lipid A is distinguished by its incorporation of significantly longer fatty acid chains (C28). This structural difference greatly diminishes the agonist activity of TLR4 and reduces the endotoxic potential of *Brucella* LPS, facilitating its evasion of immune detection [136]. Another characteristic of *Brucella* LPS that prevents recognition by TLR4 is the unique glycosylation pattern of its core oligosaccharide component [137]. The *B. abortus* wadC glycosyltransferase mutant, which possesses a disrupted LPS core but an intact O-polysaccharide and lipid A, induces robust inflammatory responses in mice and fail to replicate in DCs, leading to its targeting to lysosomal compartments [30]. Unlike wild-type *B. abortus*, the wadC mutant triggers DCs maturation and cytokine secretion through TLR4. The LPS core of the mutated wadC strain display increased binding to myeloid differentiation-2 (MD-2), the TLR4 co-receptor, enhancing intracellular signaling [30]. Notably, caspase-11 has been shown to detect cytoplasmic LPS and induce septic shock through a mechanism independent of TLR4 [138,139]. During *B. abortus* systemic infection, caspase-11 knockout mice were found to be more susceptible than wild-type animals, with fewer immune cells, such as neutrophils, macrophages, and DCs cells, recruited to the spleens. Furthermore, guanylate-binding proteins (GBPs) located on mouse chromosome 3 contribute to LPS recognition by caspase-11, promoting non-canonical inflammasome activation [140]. This mechanism, on the other hand, demonstrates the non-redundant immune system processes that detect LPS derived from pathogenic bacteria, contributing to infection control. Nevertheless, TcpB, induces the ubiquitination and degradation of inflammatory caspases −1 and −11. Hence, TcpB inhibits LPS-induced non-canonical inflammasome activation, suppressing pyroptosis and IL-1β secretion [141].

Flagellin, the monomeric building block of the bacterial flagellar filament, functions as a pathogen-associated molecular pattern (PAMP) recognized extracellularly by TLR5 [142]. Upon activation, TLR5 triggers cellular signaling pathways that activate immune cells, leading to the production and release of pro-inflammatory cytokines essential for combating bacterial infections [143]. In that context, bacterial strategies to evade flagellin detection by the innate immune system have also been identified [144]. *Brucella* evades TLR5 activation by tightly regulating the synthesis and delivery of flagellin into host cells, a key aspect of its stealth strategy to avoid detection by the innate immune system [145].

Beyond its ability to avoid innate immune receptor recognition, *Brucella* also employs mechanisms to counteract cellular signaling transduction, disrupting the immune response to facilitate its survival within the host. An example of this is the *Brucella* TIR containing protein BtpA/TcpB, which is translocated into host cells and targets the pathways activated by TLR2 and TLR4, a key host response mechanism involved in bacterial detection [53,137]. TcpB has been shown to share similarities with the TIR domain protein MyD88-adapter – like (MAL) [55]. Mechanistically, TcpB disrupts the MAL-TLR4 interaction, thereby attenuating the cellular signaling cascade [54]. Additionally, TcpB interacts with MAL, promoting its polyubiquitination and subsequent degradation via the proteasome. This process ultimately decreases the amount of phosphorylated MAL available for signaling transduction [55]. Indeed, TcpB-mediated disruption of MAL functionality enhances the survival of *Brucella* within the host [146]. Similarly, BtpA interferes with TLR2-mediated activation of the nuclear factor-κB (NF-B) pathway, which inhibits DCs maturation and function. Consequently, this impairs the production of inflammatory cytokines and may disrupt antigen presentation to T lymphocytes [53].

The Omps of *Brucella* are crucial for maintaining the integrity of the bacterial membrane and serve as key virulence factors. These proteins interfere with cellular signaling pathways, aiding in immune evasion and promoting *Brucella* pathogenesis [147,148]. A notable characteristic of Omp25 is its capacity to suppress TNF-α production in macrophage cell lines [149], a crucial cytokine for protection against persistent *Brucella* infection [150]. This virulence factor suppresses the NF-κB pathway downregulating TNF-α transcription [148]. Remarkably, Omp25 inhibits TNF-α expression by modulating microRNAs that target TNF receptor associated factor 6 (TRAF6) and Interleukin 1 Receptor Associated Kinase 1 (IRAK1), thereby negatively impacting NF-κB signaling [148]. *Brucella* Omp25 also induces the expression of the programmed cell death-1 (PD-1) receptor, which modulates microRNAs to target TAK1-binding protein 2 (TAB2) [151]. TAB2, an adaptor protein, mediates the IκB phosphorylation and degradation, leading to NF-κB release and nuclear translocation and in this manner driving inflammatory cytokine expression [152]. By disrupting this pathway, Omp25 inhibits IL-12 production via PD-1 signaling and the upregulation of specific microRNAs [151]. Moreover, Omp25 also binds to the immune receptor SLAMF1, a member of the signaling lymphocyte activation molecule family (SLAMF). This interaction inhibits NF-κB translocation in DCs, thereby reducing inflammatory cytokine secretion and cellular activation, ultimately dampening the immune response during the acute phase of infection [153]. More recently, it was demonstrated that Omp25 inhibits inflammatory cytokine production by promoting the ubiquitination and degradation of TLRs and their adaptor proteins [154]. In addition to modulating inflammatory cytokine production, Omp25 inhibits the activation of the stimulator of interferon genes (STING) pathway by suppressing the phosphorylation and nuclear translocation of interferon regulatory factor 3 (IRF3) in virus-infected macrophages. This mechanism involves the proteasomal degradation of cyclic guanosine monophosphate – adenosine monophosphate synthase (cGAS), leading to reduced cyclic GMP-AMP (cGAMP) production and subsequent suppression of type I IFN (IFN-I) production [155].

Omp31 is another important factor that contributes to maintaining the integrity of the bacterial outer membrane, and its role in *B. melitensis* virulence has been demonstrated [156]. Interesting, Omp31 is not essential for the virulence of *B. abortus*, as the gene encoding it is absent in this species due to a genomic deletion [157]. Additionally, Omp31 was associated with autophagosome formation by increasing the levels of LC3B-II and Beclin-1. Furthermore, Omp31-induced autophagy suppresses TNF-α production through the modulation of NF-κB signaling [158]. Notably, Omp31 also reduces apoptotic factors in macrophages induced by TNF-α [159]. The modulation of TNF-α production and its pro-apoptotic effects may enhance the intracellular survival of the bacteria.

*Brucella* employs a sophisticated immune evasion strategy by manipulating host microRNA pathways to impair STING-mediated innate immune signaling. The pathogen suppresses STING expression at both transcript and protein levels via post-transcriptional regulation. Specifically, *Brucella* upregulates miR-24–2, which directly targets STING mRNA for degradation. Inhibition of miR-24 or deletion of the mirn23a locus in macrophages restores STING expression and is associated with reduced bacterial replication [160].

## Unfolded protein response activation during *Brucella* infection: implications for host-pathogen interactions

The ER is a vital organelle responsible for the synthesis, folding, and processing of secretory and membrane proteins. Despite providing an optimized environment for protein folding, the ER capacity can be overwhelmed by physiological stressors, including increased protein synthesis, oxidative stress, or nutrient deprivation. This imbalance leads to the accumulation of misfolded or unfolded proteins, a condition referred to as ER stress [161]. In response, the UPR is activated as an adaptative mechanism to restore homeostasis by enhancing the protein-folding capacity, attenuating protein synthesis, and promoting the degradation of misfolded proteins [162]. This response is orchestrated by three key ER-resident sensors: IRE1, PERK, and ATF6, which activate interconnected signaling pathways regulating both adaptive and apoptotic responses to ER stress [163].

Beyond maintaining proteostasis, the UPR has emerged as a critical regulator in host-pathogen interactions [164]. During infections, the UPR acts as a double-edged sword, acting as both a target exploited by pathogens and a defense mechanism for the host. Pathogens such as viruses, bacteria, and parasites exploit the host ER machinery for replication, thereby inducing ER stress and activating the UPR. Conversely, host cells utilize UPR signaling to counteract infections by enhancing antigen presentation, promoting autophagy or inducing apoptosis to limit pathogen spread [165]. This interplay is particularly evident during *Brucella* infections, as the bacterium directly interacts with the ER to establish its replicative niche, positioning the UPR at the intersection of host-pathogen interactions.

The role of the UPR during *Brucella* infections was first suggested nearly two decades ago by an RNA interference screen that identified IRE1α as a key factor in *Brucella* pathogenesis [166]. Subsequent studies confirmed the activation of the UPR in infections caused by different *Brucella* species. For instance, *B. melitensis* activates all three primary UPR signaling pathways through the bacterial effector protein TcpB, which reorganizes ER structure and induces UPR target genes, such as BiP and CHOP. Pharmacological inhibition of the UPR reduces *B. melitensis* replication, emphasizing the role of the UPR in promoting intracellular survival [61]. Similarly, *B. abortus* triggers the UPR through a mechanism involving the bacterial second messenger cyclic di-GMP (c-di-GMP), which activates the ER-resident protein STING. STING induces the UPR and drives IFN-I production, facilitating bacterial replication [167]. Although both species activate the UPR to facilitate intracellular replication, their distinct mechanisms underscore species-specific strategies *Brucella* employs to exploit host cellular pathways to its advantage.

*Brucella* effectors play critical roles in inducing the UPR. While *virB* mutants can still activate the UPR, TcpB mutants exhibit reduced expression of UPR markers and ER structural disruption [61]. In *B. abortus*, VceC directly induces ER stress by interacting with the ER chaperone BiP, leading to IRE1α activation and pro-inflammatory responses via UPR-mediated NF-κB signaling [47]. This VceC-mediated ER stress activates the NOD1/NOD2/RIP2 signaling axis, contributing to inflammation, placentitis and abortion in mice [49]. Other effectors, such as BspC, BspG, and BspK, have also been implicated in ER stress induction, though their precise roles in *Brucella* pathogenesis remain to be fully elucidated [88].

The interplay between the UPR and immune responses during *Brucella* infection is further highlighted by the critical role of IRE1α in activating the NLRP3 inflammasome. Specifically, IRE1α facilitates ER-mitochondria communication via NLRP3, leading to mitochondrial damage and the release of mitochondrial danger-associated molecular patterns through the caspase-2-Bid signaling axis. This cascade induces mitochondrial damage, further amplifying inflammasome signaling. Remarkably, this pathway amplifies inflammation independently of ASC and involves the interaction between NLRP3 and thioredoxin-interacting protein (TXNIP) [168].

A recent study identified the T4SS effector BspI as a key modulator of the UPR during *Brucella* infection. Mechanistically, BspI selectively inhibits the kinase activity of IRE1α, thereby attenuating IRE1-mediated proinflammatory signaling cascades. This includes the suppression of NF-κB activation and downstream cytokine production, ultimately leading to a dampened inflammatory response [169].

The UPR also plays a crucial role in regulating intracellular lifecycle of *Brucella*. Specifically, the host factor YPT-interacting protein 1A (Yip1A) is essential for the biogenesis of rBCVs, a process driven by the activation of IRE1α. Disruption of Yip1A function prevents maturation of *B. abortus* into rBCVs, and confines BCVs within Lamp2-positive compartments, impairing bacterial replication [170]. Additionally, *B. melitensis* suppresses *Bloc1s1* expression through IRE1-dependent decay (RIDD), disrupting lysosomal trafficking and facilitating ER-BCV fusion [171]. The interplay between the UPR and autophagy pathways also appears to regulate the biogenesis of rBCVs. Yip1A-dependent activation of IRE1α induces the formation of replicative vacuoles, a process that also requires the autophagy proteins ATG9 and WIPI. Silencing this autophagy components disrupts rBCV formation, confining *Brucella* to early endosomal compartments [170]. Additionally, *Brucella* subverts the IRE1α-ULK1 signaling to enhance its survival and disruption of ULK1 and Beclin-1 compromises bacterial intracellular replication [172].

**Figure 1.**
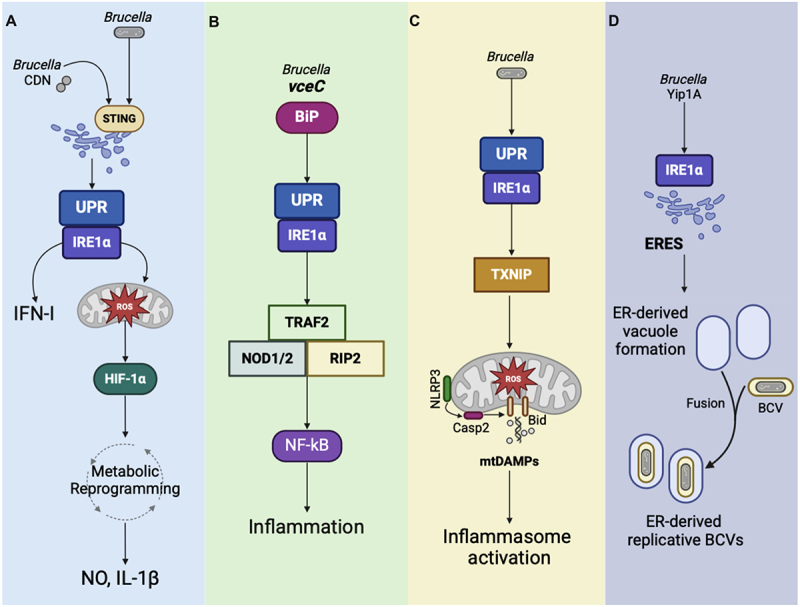
*Brucella* and the unfolded protein response. (a) *B. abortus* triggers the upr via bacterial cyclic dinucleotides (CDN) that activate sting. sting activation leads to upr induction and IFN-I production. IRE1α drives the metabolic reprograming in macrophages by stabilizing HIF-1α. IRE1α enhances mROS production, as well as NO and IL-1β release. (b) VceC directly activates the upr by interacting with the er chaperone BiP, leading to IRE1α activation. This promotes pro-inflammatory responses through the NOD1/NOD2/RIP2 axis and NF-κB signaling, contributing to inflammation. (c) IRE1α promotes ROS-dependent translocation of NLRP3 to mitochondria, where it activates the caspase-2-bid pathway, leading to mitochondrial damage and release of danger signals that engage the inflammasome via NLRP3-TXNIP interactions. (d) Yip1A facilitates the activation of IRE1α by promoting the high-order assembly of IRE1α molecules at eres under upr conditions. Upon activation, IRE1α drives the biogenesis of ER-derived vacuoles.

IRE1α also plays a crucial role in immunometabolism during *B. abortus* infection by driving the metabolic shift from oxidative phosphorylation (OXPHOS) to glycolysis. This occurs through the stabilization of hypoxia-inducible factor-1alpha (HIF-1α), a key regulator of cellular metabolism that supports the inflammatory phenotype in *Brucella*-infected macrophages [173,174]. Additionally, IRE1α enhances the production of mitochondrial reactive oxygen species (mROS), nitric oxide (NO) and the release of IL-1β all hallmarks of inflammatory macrophages. This inflammatory profile is crucial for controlling bacterial replication, as HIF-1α deficiency increases the susceptibility to *B. abortus* infection in mice [173]. Recent evidence demonstrates that *B. abortus* modulates host mitochondrial dynamics by inducing dynamin-related protein 1 (DRP1)-dependent mitochondrial fission in infected macrophages. This process is driven by IRE1α activation, effectively linking the UPR to mitochondrial remodeling. DRP1-mediated fission disrupts mitochondrial metabolism, leading to decreased mitochondrial ATP production and impaired bioenergetic capacity [175]. The key insights into the UPR during *Brucella* infections, as discussed in this review, are summarized in Figure 1.

## Subversion of host autophagy pathways by *Brucella*

Autophagy is a fundamental stress response that preserves cellular homeostasis by degrading damaged organelles, protein aggregates, and invading pathogens. While many intracellular bacteria evade xenophagy – the selective autophagic clearance of pathogens – *Brucella* has developed unique mechanisms to subvert this pathway, redirecting components of the autophagic machinery to establish a replication-permissive niche and ensure completion of its intracellular life cycle [176].

Autophagic proteins are engaged at distinct stages of the *Brucella* intracellular cycle [20]. For example, *Brucella* activates IRE1α through Yip1A, which upregulates COPII vesicle components and facilitates the conversion of BCVs into rBCVs, thereby promoting bacterial survival [170]. rBCVs formation further requires ATG9 and WIPI proteins [177]. In parallel, IRE1α activation by *B. melitensis* induces phosphorylation of ULK1, Atg9a, and Beclin-1, supporting BCV maturation while preventing lysosomal degradation [172]. Importantly, the biogenesis of autophagic aBCVs depends on ULK1, Beclin-1, ATG14L, and class III PI3-kinase, but bypasses canonical elongation factors such as ATG5, ATG7, ATG4, ATG16L1, and LC3-II. This selective exploitation of noncanonical pathways exemplifies how *Brucella* avoids xenophagic clearance while co-opting autophagy to complete its intracellular life cycle and promote intracellular dissemination [17,20].

Multiple evidence further supports the role of autophagy in *Brucella* survival. In mouse macrophages infected with *B. melitensis* LC3-II accumulation and autophagosome formation increase, whereas pharmacological inhibition of autophagy markedly reduces bacterial replication [178]. Similarly, in *B. suis*-infected macrophages, activation of the autophagy-lysosomal pathway enhances bacterial proliferation [179]. Notably, *Brucella* replicates efficiently in Atg5-deficient fibroblasts, reinforcing that canonical macroautophagy is not strictly required for intracellular survival [180].

VirB T4SS effectors further modulate autophagic flux and apoptosis, reflecting redundant strategies of host manipulation. In hepatic stellate LX-2 cells, wild-type *B. abortus* induces LC3-II and Beclin-1 accumulation, whereas *virB10* mutants fail to do so, confirming that T4SS effectors are required for autophagy induction [181]. Among these, BPE005 promotes autophagy, and deletion of *bpe005* abrogates LC3-II accumulation. Pharmacological inhibition of PI3-kinase, lysosomal proteases, or autophagosome-lysosome fusion reverses these effects, demonstrating that effector-driven autophagy is crucial for intracellular persistence [181]. Other effectors display distinct modulatory patterns: BtpB inhibits autophagy and autophagolysosome formation, while VceA selectively promotes autophagy in trophoblasts to prevent host cell death and sustain persistent infection [48,169]. BspL interacts with the ERAD pathway, delaying aBCV formation and fine-tuning replication and dissemination [95]. NyxA and NyxB interfere with the nucleolar protease SENP3, relocalizing it into structures Beclin1/PIAS3-dependent structures to create a replication-permissive environment [182]. Bioinformatics analysis of macrophages infected with ∆Omp25 *B. melitensis* identified key miRNA-mRNA networks regulating autophagy, indicating that Omp25 modulates host autophagic flux through post-transcriptional mechanisms to influence bacterial survival [183].

Recent studies reveal even more sophisticated layers of autophagy modulation. *B. abortus* triggers mitochondrial fragmentation and BNIP3L-mediated mitophagy in an iron- and HIF-1α-dependent manner. While dispensable for replication per se, mitophagy is essential for aBCV biogenesis and bacterial egress [184]. *Brucella* also modulates ferritinophagy, the selective degradation of ferritin. Secretion of the ferritin-like protein Dps sequesters labile iron and suppresses ROS, which in turn triggers compensatory NCOA4-mediated ferritinophagy, releasing iron that supports bacterial replication and ferroptotic cell death [185]. Furthermore, host genetic factors further shape autophagy responses. A CRISPR screen revealed that knockout of *DEFB103B* reduces LC3-II accumulation and enhances bacterial clearance, suggesting that certain host antimicrobial molecules paradoxically support *Brucella* persistence by sustaining autophagic processes [186].

## Type I IFN responses and host susceptibility to infection

Type I interferons (IFN-I), including IFN-α and IFN-β, are crucial cytokines in the innate immune response, playing a central role in defending against viral infections [187]. Their production is triggered by the activation of innate immune system receptors, including TLRs and cytoplasmic receptors that detect nucleic acids, particularly through the MyD88 and TRIF pathways [188]. The main sources of IFN-I are plasmacytoid dendritic cells and interstitial macrophages, activated by cytosolic receptors that recognize double-stranded RNA (dsRNA). In macrophages and DCs, TLR3 and TLR4 also induce IFN-I upon detecting dsRNA and LPS [134].

IFN-I signaling is mediated by the type I interferon receptor (IFNAR), composed of IFNAR1 and IFNAR2 subunits [189]. The binding of IFN-I to IFNAR activates the JAK-STAT pathway, promoting phosphorylation of STAT1 and STAT2, which heterodimerize and recruit IRF9 [190]. This complex translocates to the nucleus to induce the transcription of interferon-stimulated genes (ISGs). These responses include pro-apoptotic genes, chemokines, and other mediators crucial for immunity [191]. While IFN-I is traditionally associated with antiviral immunity, studies have revealed its crucial role in bacterial infections, where its production is triggered by various Gram-negative bacteria during cellular interaction or invasion [192]. The role of IFN-I in bacterial infections was first observed in investigations of *Chlamydia spp*. infections, which, despite their intracellular cycle, induce IFN-I production through TLR4 and MyD88-dependent signaling pathways [193]. Intracellular pathogens induce IFN-I through TLR4, MyD88, or cytosolic pathways, regulating both protective and detrimental responses [42]. Infections caused by pathogens such as *Listeria monocytogenes, Mycobacterium tuberculosis* (Mtb), *Salmonella Typhimurium* (SesT), and *B. abortus* demonstrate that sensors like STING, cGAS, RIG-I, and NOD2 are involved in IFN-I production. This response, by inducing apoptosis of immune cells, suppressing inflammatory cytokines (such as IL-1β and IL-18), and reducing neutrophil recruitment, ultimately favors bacterial persistence and replication [194–197].

In tuberculosis, active human disease is consistently associated with the induction of IFN-I [198]. Evidence that IFN-I exacerbates tuberculosis in humans comes from observations that viral infections are associated with worsened outcomes in Mtb infections. Recently, Ji *et al* [199]., identified Sp140 as a transcriptional regulator of IFN-I that controls Mtb susceptibility. This research group suggests a model of tuberculosis pathogenesis in which IFN-I drives an initial loss of bacterial control, possibly by impairing IFN-γ responses, that, in turn, initiates a positive feedback loop of NET production and IFN-I expression by pDCs, leading to uncontrolled bacterial replication and active tuberculosis disease [196].

In the context of *Brucella* infections, the pioneering study by Huang *et al* [200]., investigated the relationship between *B. abortus* and IFN-I, demonstrating that wild-type mice had detectable levels of IFN-α in their serum just three hours after administration of heat-killed *B. abortus*. In contrast, TLR9-deficient mice showed significantly reduced levels of IFN-α, indicating that IFN-I induction by *B. abortus* depends on a TLR9-mediated pathway. Additionally, IFNAR-deficient mice showed a reduced bacterial load in the spleen and higher production of inflammatory mediators, such as IFN-γ and NO, compared to wild-type controls.

Our group has been investigating the interaction between IFN-I and *B. abortus* for several years. We demonstrated that *Brucella* infection induces IFN-I production in macrophages and splenocytes mediated by MyD88-dependent pathways, IRF3 signaling and intracellular sensors such as STING and ZBP1 [167,201]. We observed that IFN-I has a detrimental effect on the host by suppressing the production of inflammatory cytokines like IL-1β and IFN-γ, while also modulating pro-apoptotic genes such as TRAIL. IFN-I-deficient mice exhibited lower bacterial load and reduced apoptosis, suggesting that IFN-I favors *B. abortus* survival [197]. Similar findings have been observed with *Listeria monocytogenes* [202,203].

Recently, we demonstrated that *B. abortus*-induced UPR has an important role in inducing IFN-β responses and is linked to the production of several molecules associated with the IFN-I pathway [167]. Additionally, macrophages treated with a mouse recombinant IFN-β (rIFN-β) showed enhanced *XBP1*(s) expression, a downstream target of IRE1α activation, and enhanced number of *B. abortus* CFU *in vitro* [167]. Collectively, these findings demonstrate that treatment with rIFN-β enhances *B. abortus* replication, underscoring the pivotal role of IFN-β in *B. abortus* replication and survival.

We recently identified a previously unrecognized role for DRP1 in regulating IFN-I production and signaling during *Brucella* infection. Mechanistically, DRP1-dependent mitochondrial fission facilitates the release of mitochondrial DNA into the cytosol, possibly serving as a potent trigger for IFN-I responses. This process establishes a link between mitochondrial dynamics and innate immune signaling, highlighting how *B. abortus* can modulate host pathways through targeted remodeling of mitochondrial morphology [175].

Furthermore, *Brucella* Omp25 specifically targets the cGAS-STING signaling pathway, inhibiting its production of IFN-β by promoting cGAS degradation in macrophages via the ubiquitin-proteasome-dependent pathway upon DNA virus infection or DNA stimulation [155].

IFN-I is unquestionably crucial for the immune response. However, in some cases, it can promote pathogen evasion and reduce the effectiveness of the immune response, compromising bacterial infection control. This ambiguous effect highlights the need for careful regulation of IFN-I signaling to ensure an effective response against a variety of pathogens.

## *Brucella* and host metabolism: a battle for resources

Cellular metabolism is crucial in regulating innate immune activation, as metabolic changes in immune cells often dictate pro-inflammatory or anti-inflammatory responses [204]. Immune cells undergo metabolic reprogramming to meet the energy and biosynthetic demands necessary for their activation and effector functions. During an inflammatory response, macrophages and DCs primarily shift toward glycolysis, enabling the rapid production of ATP and biosynthetic intermediates vital for cytokine secretion and pathogen elimination. Notably, cells may prioritize glycolysis for ATP production even in the presence of sufficient oxygen, a phenomenon known as the Warburg effect (aerobic glycolysis) [205]. This metabolic reprogramming impacts the production of key metabolites such as lactate, succinate, and itaconate, which act as signaling molecules regulating immune responses [206].

*Brucella* exploits these metabolic adaptations to establish and sustain chronic infection. Key *Brucella* genes essential for persistence are associated with its ability to utilize diverse nutrient sources, suggesting that the bacterium has evolved mechanisms to exploit both immune responses and limited nutrient availability within host cells [137]. The adaptation of *Brucella* to the macrophage environment is particularly significant, as it adjusts to distinct metabolic pathways associated with pro-inflammatory (M1) and anti-inflammatory (M2) macrophages. M1 macrophages rely primarily on aerobic glycolysis, producing lactate while utilizing the tricarboxylic acid cycle (TCA) cycle to generate citrate and succinate, which are essential for fatty acid metabolism and HIF-1α stabilization. These processes enhance the transcription of pro-inflammatory and glycolytic genes while promoting the production of NO and ROS. In contrast, M2 macrophages depend on fatty acid oxidation and OXPHOS to meet their metabolic needs [207].

Inflammatory M1 macrophages predominate during the early stages of *Brucella* infection and are characterized by increased nitric oxide synthase 2 (NOS2) expression and other pro-inflammatory markers, while anti-inflammatory M2 macrophages become more prominent during the chronic phase [173]. Since NOS2 expression and pro-inflammatory cytokines are crucial for controlling *B. abortus* growth [208,209], M1 macrophages are expected to manage the infection more effectively than the M2 phenotype. Indeed, NF-κB signaling is crucial to promote M1 polarization, which significantly reduces *B. abortus* survival in a macrophage cell line. In this scenario, the glutaminase (Gls) enzyme, a key NF-κB target, enhances the expression of M1-associated markers, while its inhibition promotes M2 polarization and facilitates bacterial replication [210].

Furthermore, during *Brucella* infection, STING promotes HIF-1α stabilization, reprogramming macrophage metabolism from OXPHOS to glycolysis, enhancing NO production and inflammasome activation. Likewise, the mitochondrial function shifts in macrophages from ATP synthesis to ROS production. These processes are critical for controlling *Brucella* persistence [173]. However, during chronic infection, *Brucella* circumvents these defenses by preferentially replicating in M2-like macrophages, where it benefits from increased glucose accumulation driven by the activity of peroxisome proliferator-activated receptor γ (PPARγ). M2-like macrophages rely on fatty acid β-oxidation for ATP production in a PPARγ-dependent manner, leading to higher intracellular glucose accumulation compared to M1-like macrophages. This glucose accumulation supports *Brucella* growth, and the inactivation of the bacterial glucose transporter gluP significantly reduces bacterial survival in macrophages [211]. The enhanced survival of *B. abortus* in M2-like macrophages is also linked to a shift in host arginine metabolism from NO production to polyamines synthesis, driven by arginase-1 expression. Inhibiting polyamine synthesis or inactivating the putrescine transporter (*potIHGF*) reduces *B. abortus* persistence in M2-like macrophage, highlighting the importance of polyamine metabolism in sustaining chronic infection [212]. Another example of adaptation to the intracellular niche is observed in *B. abortus*-infected THP-1 cells. *Brucella* disrupts mitochondrial function and alters the localization of mitochondria, changing the metabolism of amino acids that feed the TCA. This metabolic shift reduces amino acid catabolism and increases lactate production, allowing the bacteria to utilize host-derived amino acids and lactate as energy sources to support its intracellular persistence [213].

**Figure 2.**
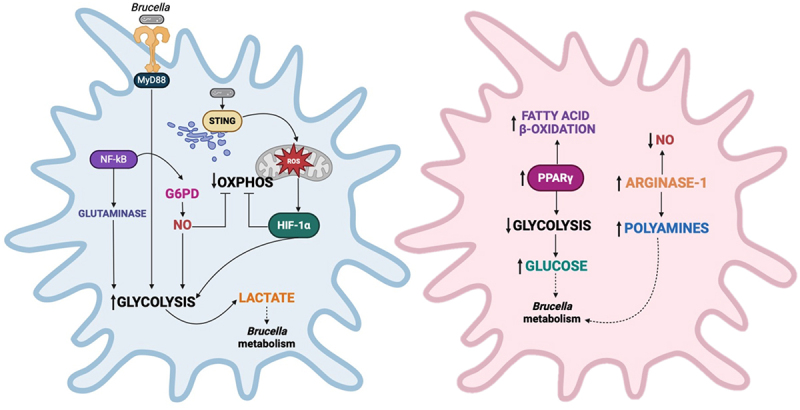
Overview of immunometabolism in *Brucella*-infected macrophages. Inflammatory M1 macrophages (left) predominate during the early stages of *Brucella* infection, playing a critical role in infection control. The TLR/MyD88 and NF-κB signaling pathways promote glycolysis and glutaminase-driven expression of pro-inflammatory markers. NF-κB upregulates G6PD, which facilitates the shift to glycolysis by regulating no production. sting activation enhances mROS production, contributing to HIF-1α stabilization, which promotes glycolysis and suppresses OXPHOS. This metabolic reprogramming increases glucose consumption to meet energy demands, resulting in elevated lactate levels, which *Brucella* can exploit as an energy source. During chronic infection, anti-inflammatory M2 macrophages (right) support *Brucella* persistence. PPARγ activation promotes fatty acid β-oxidation, reducing host glucose utilization and increasing intracellular glucose availability for bacterial replication. Arginase-1 shifts arginine metabolism from no production to polyamines synthesis, providing additional nutrients for the bacteria. Moreover, reduced no and suppressed pro-inflammatory cytokine production contribute to infection chronicity, allowing the pathogen to establish a long-term intracellular niche.

*Brucella* also regulates glucose metabolism by activating NF-κB to modulate glucose-6-phosphate dehydrogenase (G6PD) expression, which influences the switch to glycolysis by regulating NO levels during *Brucella* infection [214]. Additionally, MyD88 contributes to macrophage glycolysis in response to *B. melitensis*, by inducing metabolic changes that control key metabolites, including itaconate, a metabolite with antibacterial effects against *Brucella* [215]. Moreover, BtpA and BtpB, through their NAD^+^ hydrolase activity, deplete NAD^+^ levels in host cells, modulating host metabolism. Both proteins induce growth arrest in yeast cells, a process associated with actin depolymerization and reduced kinase activity, suggesting impaired energy metabolism linked to decreased ATP and NAD^+^ levels [62]. A summary of the data presented in this section is provided in Figure 2.

## Conclusions and future perspectives

*Brucella* species have evolved highly specialized mechanisms to manipulate host immune responses and metabolic pathways, facilitating their adaptation to the host environment. This review has highlighted the intricate strategies by which *Brucella* modulates immune signaling networks, particularly IFN-I responses and immunometabolism, to evade detection and suppress antimicrobial defenses. Additionally, the ability to exploit the UPR underscores their sophisticated adaptation to intracellular stress, promoting long-term persistence within host cells.

Despite significant progress in understanding *Brucella* pathogenesis, critical knowledge gaps remain regarding the precise molecular interplay between *Brucella* effectors and host immune regulators, as well as the metabolic rewiring strategies that sustain chronic infection. Future research should focus on elucidating the regulatory circuits governing *Brucella* adaptive responses and identifying metabolic vulnerabilities that could be exploited as therapeutic targets. Furthermore, investigating the crosstalk between *Brucella*-induced UPR activation and immune evasion mechanisms may reveal novel potential targets for host-directed therapies.

Advances in single-cell RNA sequencing (scRNA-seq) have enabled the analysis of transcriptional heterogeneity during infections. In the bacterial context, methods such as MATQ-seq, microSPLiT, and PETRI-seq allow unbiased gene expression profiling in individual prokaryotic cells, revealing phenotypic states and transcriptional programs associated with persistence and immune evasion [216]. These transcriptomic technologies are increasingly being applied to dissect host-pathogen interactions in *Brucella* infection models. A dual RNA-seq study in murine macrophages simultaneously profiled both *B. abortus* and host gene expression, revealing how the bacterium adapts its metabolic and virulence gene programs to the intracellular environment while modulating host innate immune pathways [217]. Complementarily, a recent single-cell RNA-seq study in human brucellosis patients identified immune phase-specific signatures, including functional exhaustion and impaired antigen presentation in chronic stages of disease [218]. These findings highlight the value of transcriptomic approaches in uncovering key virulence strategies and host responses, offering new directions for future research and therapeutic development.

Alongside transcriptomic innovations, artificial intelligence (AI) and machine learning (ML) approaches are being progressively explored in *Brucella* research. Recent studies have applied ML algorithms to tasks such as species classification based on MALDI-TOF MS spectra, with promising levels of accuracy [219,220] and to reverse vaccinology pipelines aimed at optimizing antigen selection for vaccine development [221–223]. In addition to these applications, AI-based methods have shown promise in analyzing multi-omics and functional genomics data in the context of *Brucella* [224]. Models such as Vaxign ML, for example, have been successfully employed to prioritize multi-epitope vaccine candidates against *Brucella*, based on genomic and proteomic features [224–226]. Although still in early stages, the integration of AI-guided analyses with experimental techniques such as scRNA-seq and dual RNA-seq holds promise for uncovering novel virulence mechanisms, predicting molecular markers of persistence, and refining the selection of therapeutic and vaccine targets.

Beyond pathogenesis, there is an urgent need for more effective diagnostic tools and next-generation vaccines with improved safety and immunogenicity. Current vaccines, while partially protective, present limitations such as residual virulence and inconsistent efficacy across host species. Integrating advanced approaches in systems biology, genomics, and immunology may facilitate the design of targeted therapeutic interventions and optimized vaccine formulations.

Among next-generation vaccine platforms, mRNA-based vaccines are gaining attention as a promising alternative to overcome the limitations of conventional *Brucella* vaccines, particularly in the context of persistent infection [222]. In contrast to attenuated or inactivated bacterial vaccines, which retain residual virulence and interfere with standard serological diagnostics [227,228], mRNA vaccines offer several advantages, including rapid development, strong immunogenicity, and flexible epitope targeting [229]. Recent *in silico* studies have designed multi-epitope mRNA vaccine constructs targeting antigens such as BtuB, LptD, and outer membrane proteins of *B. melitensis*, showing favorable molecular stability, antigenicity, and MHC-binding affinity [229,230]. These formulations are predicted to induce robust CD4^+^ and CD8^+^ T cell responses with a Th1 profile, which is essential for the clearance of intracellular pathogens [231]. As these candidates progress toward preclinical validation, they are expected to complement systems-level immunological insights and pave the way for more precise therapeutic strategies against chronic *Brucella* infection.

A deeper understanding of *Brucella* immunomodulatory strategies, metabolic adaptations, and virulence determinants will be instrumental in advancing targeted intervention strategies. This knowledge will not only enhance our ability to combat *Brucella* infections but also provide broader insights into host-pathogen interactions and chronic bacterial persistence mechanisms.

## Data Availability

Data sharing is not applicable to this article as no new data were created or analyzed in this study.

## References

[1] WHO. Brucellosis in humans and animals. Geneva, Switzerland: World Health Organization; 2006.

[2] Franc KA, Krecek RC, Häsler BN, et al. Brucellosis remains a neglected disease in the developing world: a call for interdisciplinary action. BMC Public Health. 2018;18(1):125. doi: 10.1186/s12889-017-5016-y 29325516 PMC5765637

[3] Jiao H, Zhou Z, Li B, et al. The mechanism of facultative intracellular parasitism of *Brucella*. Int J Mol Sci. 2021;22(7):3673. doi: 10.3390/ijms22073673 33916050 PMC8036852

[4] Percin D. Microbiology of *Brucella*. Recent Pat Antiinfect Drug Discov. 2013;8(1):13–24. doi: 10.2174/157489113805290683 22812617

[5] Corbel MJ. Brucellosis: an overview. Emerg Infect Dis. 1997;3(2):213–221. doi: 10.3201/eid0302.970219 9204307 PMC2627605

[6] Carvalho TP, Silva LAD, Castanheira TLL, et al. Cell and tissue tropism of *Brucella* spp. Infect Immun. 2023;91(5):e0006223. doi: 10.1128/iai.00062-23 37129522 PMC10187126

[7] Qureshi KA, Parvez A, Fahmy NA, et al. Brucellosis: epidemiology, pathogenesis, diagnosis and treatment-a comprehensive review. Ann Med. 2023;55(2):2295398. doi: 10.1080/07853890.2023.2295398 38165919 PMC10769134

[8] Laine CG, Johnson VE, Scott HM, et al. Global estimate of human brucellosis incidence. Emerg Infect Dis. 2023;29(9):1789–1797. doi: 10.3201/eid2909.230052 37610167 PMC10461652

[9] Atluri VL, Xavier MN, de Jong MF, et al. Interactions of the human pathogenic *Brucella* species with their hosts. Annu Rev Microbiol. 2011;65(1):523–541. doi: 10.1146/annurev-micro-090110-102905 21939378 PMC13363517

[10] Pappas G, Akritidis N, Bosilkovski M, et al. Brucellosis. N Engl J Med. 2005;352(22):2325–2336. doi: 10.1056/NEJMra050570 15930423

[11] Pourbagher A, Pourbagher MA, Savas L, et al. Epidemiologic, clinical, and imaging findings in brucellosis patients with osteoarticular involvement. AJR Am J Roentgenol. 2006;187(4):873–880. doi: 10.2214/AJR.05.1088 16985128

[12] Baldi PC, Giambartolomei GH. Pathogenesis and pathobiology of zoonotic brucellosis in humans. Rev Sci Tech. 2013;32(1):117–125. doi: 10.20506/rst.32.1.2192 23837370

[13] Elbehiry A, Aldubaib M, Marzouk E, et al. The development of diagnostic and vaccine strategies for early detection and control of human brucellosis, particularly in endemic areas. Vaccines (Basel). 2023;11(3):654. doi: 10.3390/vaccines11030654 36992237 PMC10054502

[14] Maduranga S, Valencia BM, Li X, et al. A systematic review and meta-analysis of comparative clinical studies on antibiotic treatment of brucellosis. Sci Rep. 2024;14(1):19037. doi: 10.1038/s41598-024-69669-w 39152180 PMC11329684

[15] Archambaud C, Salcedo SP, Lelouard H, et al. Contrasting roles of macrophages and dendritic cells in controlling initial pulmonary *Brucella* infection. Eur J Immunol. 2010;40(12):3458–3471. doi: 10.1002/eji.201040497 21108467

[16] Celli J. The changing nature of the *Brucella*-containing vacuole. Cell Microbiol. 2015;17(7):951–958. doi: 10.1111/cmi.12452 25916795 PMC4478208

[17] Celli J. The intracellular life cycle of *Brucella* spp. Microbiol Spectr. 2019;7(2). doi: 10.1128/microbiolspec.BAI-0006-2019 PMC6448592 30848234

[18] Starr T, Ng TW, Wehrly TD, et al. *Brucella* intracellular replication requires trafficking through the late endosomal/lysosomal compartment. Traffic. 2008;9(5):678–694. doi: 10.1111/j.1600-0854.2008.00718.x 18266913

[19] Comerci DJ, Martinez-Lorenzo MJ, Sieira R, et al. Essential role of the VirB machinery in the maturation of the *Brucella* abortus-containing vacuole. Cell Microbiol. 2001;3(3):159–168. doi: 10.1046/j.1462-5822.2001.00102.x 11260139

[20] Starr T, Child R, Wehrly T, et al. Selective subversion of autophagy complexes facilitates completion of the *Brucella* intracellular cycle. Cell Host Microbe. 2012;11(1):33–45. doi: 10.1016/j.chom.2011.12.002 22264511 PMC3266535

[21] Sangari FJ, Aguero J. Molecular basis of *Brucella* pathogenicity: an update. Microbiologia. 1996;12(2):207–218. 8767705

[22] Tsolis RM, Young GM, Solnick JV, et al. From bench to bedside: stealth of enteroinvasive pathogens. Nat Rev Microbiol. 2008;6(12):883–892. doi: 10.1038/nrmicro2012 18955984

[23] Porte F, Naroeni A, Ouahrani-Bettache S, et al. Role of the *Brucella* suis lipopolysaccharide O antigen in phagosomal genesis and in inhibition of phagosome-lysosome fusion in murine macrophages. Infect Immun. 2003;71(3):1481–1490. doi: 10.1128/IAI.71.3.1481-1490.2003 12595466 PMC148865

[24] Watarai M, Makino S-I, Michikawa M, et al. Macrophage plasma membrane cholesterol contributes to *Brucella* abortus infection of mice. Infect Immun. 2002;70(9):4818–4825. doi: 10.1128/IAI.70.9.4818-4825.2002 12183525 PMC128274

[25] Forestier C, Deleuil F, Lapaque N, et al. *Brucella* abortus lipopolysaccharide in murine peritoneal macrophages acts as a down-regulator of T cell activation. J Immunol. 2000;165(9):5202–5210. doi: 10.4049/jimmunol.165.9.5202 11046053

[26] Roop RM, Barton IS, Hopersberger D, et al. Uncovering the hidden credentials of *Brucella* virulence. Microbiol Mol Biol Rev. 2021;85(1). doi: 10.1128/MMBR.00021-19 PMC8549849 33568459

[27] Chen F, He Y. Caspase-2 mediated apoptotic and necrotic murine macrophage cell death induced by rough *Brucella* abortus. PLOS ONE. 2009;4(8):e6830. doi: 10.1371/journal.pone.0006830 19714247 PMC2729395

[28] Fontana C, Conde-Álvarez R, Ståhle J, et al. Structural studies of lipopolysaccharide-defective mutants from *Brucella* melitensis identify a core oligosaccharide critical in virulence. J Biol Chem. 2016;291(14):7727–7741. doi: 10.1074/jbc.M115.701540 26867577 PMC4817197

[29] Salvador-Bescos M, Gil-Ramírez Y, Zúñiga-Ripa A, et al. Wadd, a new *Brucella* lipopolysaccharide core glycosyltransferase identified by genomic search and phenotypic characterization. Front Microbiol. 2018;9:2293. doi: 10.3389/fmicb.2018.02293 30319590 PMC6171495

[30] Conde-Alvarez R, Arce-Gorvel V, Iriarte M, et al. The lipopolysaccharide core of *Brucella* abortus acts as a shield against innate immunity recognition. PLOS Pathog. 2012;8(5):e1002675. doi: 10.1371/journal.ppat.1002675 22589715 PMC3349745

[31] Gil-Ramirez Y, Conde-Álvarez R, Palacios-Chaves L, et al. The identification of wadB, a new glycosyltransferase gene, confirms the branched structure and the role in virulence of the lipopolysaccharide core of *Brucella* abortus. Microb Pathog. 2014;73:53–59. doi: 10.1016/j.micpath.2014.06.002 24927935

[32] O’Callaghan D, Cazevieille C, Allardet‐Servent A, et al. A homologue of the Agrobacterium tumefaciens VirB and Bordetella pertussis ptl type IV secretion systems is essential for intracellular survival of *Brucella* suis. Mol Microbiol. 1999;33(6):1210–1220. doi: 10.1046/j.1365-2958.1999.01569.x 10510235

[33] Porte F, Liautard JP, Kohler S. Early acidification of phagosomes containing *Brucella* suis is essential for intracellular survival in murine macrophages. Infect Immun. 1999;67(8):4041–4047. doi: 10.1128/IAI.67.8.4041-4047.1999 10417172 PMC96697

[34] Arocena GM, Sieira R, Comerci DJ, et al. Identification of the quorum-sensing target DNA sequence and N-acyl homoserine lactone responsiveness of the *Brucella* abortus virB promoter. J Bacteriol. 2010;192(13):3434–3440. doi: 10.1128/JB.00232-10 20400542 PMC2897657

[35] Sieira R, Comerci DJ, Pietrasanta LI, et al. Integration host factor is involved in transcriptional regulation of the *Brucella* abortus virB operon. Mol Microbiol. 2004;54(3):808–822. doi: 10.1111/j.1365-2958.2004.04316.x 15491369

[36] Rambow-Larsen AA, Rajashekara G, Petersen E, et al. Putative quorum-sensing regulator BlxR of *Brucella* melitensis regulates virulence factors including the type IV secretion system and flagella. J Bacteriol. 2008;190(9):3274–3282. doi: 10.1128/JB.01915-07 18310341 PMC2347389

[37] Caswell CC, Gaines JM, Roop RM. The RNA chaperone Hfq independently coordinates expression of the VirB type IV secretion system and the LuxR-type regulator BabR in *Brucella* abortus 2308. J Bacteriol. 2012;194(1):3–14. doi: 10.1128/JB.05623-11 22020650 PMC3256608

[38] Hong PC, Tsolis RM, Ficht TA. Identification of genes required for chronic persistence of *Brucella* abortus in mice. Infect Immun. 2000;68(7):4102–4107. doi: 10.1128/IAI.68.7.4102-4107.2000 10858227 PMC101704

[39] Xiong X, Li B, Zhou Z, et al. The VirB system plays a crucial role in *Brucella* intracellular infection. Int J Mol Sci. 2021;22(24):13637. doi: 10.3390/ijms222413637 34948430 PMC8707931

[40] Celli J, de Chastellier C, Franchini D-M, et al. *Brucella* evades macrophage killing via VirB-dependent sustained interactions with the endoplasmic reticulum. J Exp Med. 2003;198(4):545–556. doi: 10.1084/jem.20030088 12925673 PMC2194179

[41] Pizarro-Cerda J, Moreno E, Sanguedolce V, et al. Virulent *Brucella* abortus prevents lysosome fusion and is distributed within autophagosome-like compartments. Infect Immun. 1998;66(5):2387–2392. doi: 10.1128/IAI.66.5.2387-2392.1998 9573138 PMC108212

[42] Roux CM, Rolán HG, Santos RL, et al. *Brucella* requires a functional type IV secretion system to elicit innate immune responses in mice. Cell Microbiol. 2007;9(7):1851–1869. doi: 10.1111/j.1462-5822.2007.00922.x 17441987

[43] Rolan HG, Tsolis RM. Inactivation of the type IV secretion system reduces the Th1 polarization of the immune response to *Brucella* abortus infection. Infect Immun. 2008;76(7):3207–3213. doi: 10.1128/IAI.00203-08 18458071 PMC2446720

[44] Li Z, Wang S, Han J, et al. Expression of cytokine and apoptosis-associated genes in mice bone marrow-derived macrophages stimulated with *Brucella* recombinant type IV secretion effectors. Cytokine. 2024;182:156711. doi: 10.1016/j.cyto.2024.156711 39094437

[45] Yin Y, Tian M, Zhang G, et al. Identification of *Brucella* RS15060 as a novel type IV secretion system effector associated with bacterial virulence. Vet Res. 2024;55(1):168. doi: 10.1186/s13567-024-01417-4 39696601 PMC11654162

[46] Gimenez A, Del Giudice MG, López PV, et al. *Brucella* NpeA is a secreted type IV effector containing an N-WASP-binding short linear motif that promotes niche formation. MBio. 2024;15(7):e0072624. doi: 10.1128/mbio.00726-24 38847540 PMC11253601

[47] de Jong MF, Starr T, Winter MG, et al. Sensing of bacterial type IV secretion via the unfolded protein response. MBio. 2013;4(1):e00418–12. doi: 10.1128/mBio.00418-12 23422410 PMC3624511

[48] Zhang J, Li M, Li Z, et al. Deletion of the type IV secretion system effector VceA promotes autophagy and inhibits apoptosis in *Brucella*-infected human trophoblast cells. Curr Microbiol. 2019;76(4):510–519. doi: 10.1007/s00284-019-01651-6 30805699

[49] Keestra-Gounder AM, Byndloss MX, Seyffert N, et al. NOD1 and NOD2 signalling links ER stress with inflammation. Nature. 2016;532(7599):394–397. doi: 10.1038/nature17631 27007849 PMC4869892

[50] Byndloss MX, Tsai AY, Walker GT, et al. *Brucella* abortus infection of placental trophoblasts triggers endoplasmic reticulum stress-mediated cell death and fetal loss via type IV secretion system-dependent activation of CHOP. MBio. 2019;10(4). doi: 10.1128/mBio.01538-19 PMC6650558 31337727

[51] Zhi F, Zhou D, Bai F, et al. Vcec mediated IRE1 pathway and inhibited CHOP-induced apoptosis to support *Brucella* replication in goat trophoblast cells. Int J Mol Sci. 2019;20(17):4104. doi: 10.3390/ijms20174104 31443507 PMC6747397

[52] Radhakrishnan GK, Yu Q, Harms JS, et al. *Brucella* TIR domain-containing protein mimics properties of the Toll-like receptor adaptor protein TIRAP. J Biol Chem. 2009;284(15):9892–9898. doi: 10.1074/jbc.M805458200 19196716 PMC2665112

[53] Salcedo SP, Marchesini MI, Lelouard H, et al. *Brucella* control of dendritic cell maturation is dependent on the TIR-containing protein Btp1. PLOS Pathog. 2008;4(2):e21. doi: 10.1371/journal.ppat.0040021 18266466 PMC2233671

[54] Saqib U, Baig MS. Scaffolding role of TcpB in disrupting TLR4-Mal interactions: three to tango. J Cell Biochem. 2019;120(3):3455–3458. doi: 10.1002/jcb.27619 30242887

[55] Sengupta D, Koblansky A, Gaines J, et al. Subversion of innate immune responses by *Brucella* through the targeted degradation of the TLR signaling adapter, Mal. J Immunol. 2010;184(2):956–964. doi: 10.4049/jimmunol.0902008 20018612 PMC3644118

[56] Salcedo SP, Marchesini MI, Degos C, et al. BtpB, a novel *Brucella* TIR-containing effector protein with immune modulatory functions. Front Cell Infect Microbiol. 2013;3:28. doi: 10.3389/fcimb.2013.00028 23847770 PMC3703528

[57] Li J, Qi L, Diao Z, et al. *Brucella* BtpB manipulates apoptosis and autophagic flux in RAW264.7 cells. Int J Mol Sci. 2022;23(22):14439. doi: 10.3390/ijms232214439 36430916 PMC9693124

[58] Durward M, Radhakrishnan G, Harms J, et al. Active evasion of CTL mediated killing and low quality responding CD8+ T cells contribute to persistence of brucellosis. PLOS ONE. 2012;7(4):e34925. doi: 10.1371/journal.pone.0034925 22558103 PMC3338818

[59] Radhakrishnan GK, Harms JS, Splitter GA. Modulation of microtubule dynamics by a TIR domain protein from the intracellular pathogen *Brucella* melitensis. Biochem J. 2011;439(1):79–83. doi: 10.1042/BJ20110577 21692747 PMC3513345

[60] Alves-Silva J, Tavares IP, Guimarães ES, et al. Modulation of microtubule dynamics affects *Brucella* abortus intracellular survival, pathogen-containing vacuole maturation, and pro-inflammatory cytokine production in infected macrophages. Front Microbiol. 2017;8:2217. doi: 10.3389/fmicb.2017.02217 29184543 PMC5694624

[61] Smith JA, Khan M, Magnani DD, et al. *Brucella* induces an unfolded protein response via TcpB that supports intracellular replication in macrophages. PLOS Pathog. 2013;9(12):e1003785. doi: 10.1371/journal.ppat.1003785 24339776 PMC3855547

[62] Coronas-Serna JM, Louche A, Rodríguez-Escudero M, et al. The TIR-domain containing effectors BtpA and BtpB from *Brucella* abortus impact NAD metabolism. PLOS Pathog. 2020;16(4):e1007979. doi: 10.1371/journal.ppat.1007979 32298382 PMC7188309

[63] Sola-Landa A, Pizarro‐Cerdá J, Grilló M-J, et al. A two-component regulatory system playing a critical role in plant pathogens and endosymbionts is present in *Brucella* abortus and controls cell invasion and virulence. Mol Microbiol. 1998;29(1):125–138. doi: 10.1046/j.1365-2958.1998.00913.x 9701808

[64] Rivas-Solano O, Van der Henst M, Castillo-Zeledón A, et al. The regulon of *Brucella* abortus two-component system BvrR/BvrS reveals the coordination of metabolic pathways required for intracellular life. PLOS ONE. 2022;17(9):e0274397. doi: 10.1371/journal.pone.0274397 36129877 PMC9491525

[65] Altamirano-Silva P, Meza-Torres J, Castillo-Zeledón A, et al. *Brucella* abortus senses the intracellular environment through the BvrR/BvrS two-component system, which allows B. abortus to adapt to its replicative niche. Infect Immun. 2018;86(4). doi: 10.1128/IAI.00713-17 PMC5865028 29378792

[66] Altamirano-Silva P, Cordero-Serrano M, Méndez-Montoya J, et al. Intracellular passage triggers a molecular response in *Brucella* abortus that increases its infectiousness. Infect Immun. 2021;89(7):e0000421. doi: 10.1128/IAI.00004-21 33820813 PMC8373234

[67] Guzman-Verri C, Manterola L, Sola-Landa A, et al. The two-component system BvrR/BvrS essential for *Brucella* abortus virulence regulates the expression of outer membrane proteins with counterparts in members of the Rhizobiaceae. Proc Natl Acad Sci USA. 2002;99(19):12375–12380. doi: 10.1073/pnas.192439399 12218183 PMC129452

[68] Tibor A, Wansard V, Bielartz V, et al. Effect of omp10 or omp19 deletion on *Brucella* abortus outer membrane properties and virulence in mice. Infect Immun. 2002;70(10):5540–5546. doi: 10.1128/IAI.70.10.5540-5546.2002 12228280 PMC128365

[69] Zhang L, Bai J, Li L, et al. The role of outer membrane protein 16 in *Brucella* pathogenesis, vaccine development, and diagnostic applications. Vet Sci. 2025;12(7):605. doi: 10.3390/vetsci12070605 40711265 PMC12298138

[70] Zhi F, Zhou D, Li J, et al. Omp16, a conserved peptidoglycan-associated lipoprotein, is involved in *Brucella* virulence in vitro. J Microbiol. 2020;58(9):793–804. doi: 10.1007/s12275-020-0144-y 32870485

[71] Barrionuevo P, Cassataro J, Delpino MV, et al. *Brucella* abortus inhibits major histocompatibility complex class II expression and antigen processing through interleukin-6 secretion via Toll-like receptor 2. Infect Immun. 2008;76(1):250–262. doi: 10.1128/IAI.00949-07 17984211 PMC2223644

[72] Barrionuevo P, Giambartolomei GH. Inhibition of antigen presentation by *Brucella*: many more than many ways. Microbes Infect. 2019;21(3–4):136–142. doi: 10.1016/j.micinf.2018.12.004 30677519

[73] Pasquevich KA, Carabajal MV, Guaimas FF, et al. Omp19 enables *Brucella* abortus to evade the antimicrobial activity from host’s proteolytic defense system. Front Immunol. 2019;10:1436. doi: 10.3389/fimmu.2019.01436 31297115 PMC6607954

[74] Edmonds MD, Cloeckaert A, Booth NJ, et al. Attenuation of a *Brucella* abortus mutant lacking a major 25 kDa outer membrane protein in cattle. Am J Vet Res. 2001;62(9):1461–1466. doi: 10.2460/ajvr.2001.62.1461 11560278

[75] Alakavuklar MA, Fiebig A, Crosson S. The *Brucella* cell envelope. Annu Rev Microbiol. 2023;77(1):233–253. doi: 10.1146/annurev-micro-032521-013159 37104660 PMC10787603

[76] Malinverni JC, Silhavy TJ. Assembly of outer membrane beta-barrel proteins: the BAM complex. EcoSal Plus. 2011;4(2). doi: 10.1128/ecosalplus.4.3.8 PMC4231818 26442509

[77] Herrou J, Willett JW, Fiebig A, et al. *Brucella* periplasmic protein EipB is a molecular determinant of cell envelope integrity and virulence. J Bacteriol. 2019;201(12). doi: 10.1128/JB.00134-19 PMC6531618 30936371

[78] Inon de Iannino N, Briones G, Tolmasky M, et al. Molecular cloning and characterization of cgs, the *Brucella* abortus cyclic beta(1–2) glucan synthetase gene: genetic complementation of Rhizobium meliloti ndvB and Agrobacterium tumefaciens chvB mutants. J Bacteriol. 1998;180(17):4392–4400. doi: 10.1128/JB.180.17.4392-4400.1998 9721274 PMC107446

[79] Breedveld MW, Miller KJ. Cyclic beta-glucans of members of the family Rhizobiaceae. Microbiol Rev. 1994;58(2):145–161. doi: 10.1128/mr.58.2.145-161.1994 8078434 PMC372960

[80] Briones G, de Lannino NI, Steinberg M, et al. Periplasmic cyclic 1,2-beta-glucan in *Brucella* spp. is not osmoregulated. Microbiol (read). 1997;143(4):1115–1124. doi: 10.1099/00221287-143-4-1115 9141674

[81] Roset MS, Ibañez AE, de Souza Filho JA, et al. *Brucella* cyclic beta-1,2-glucan plays a critical role in the induction of splenomegaly in mice. PLOS ONE. 2014;9(7):e101279. doi: 10.1371/journal.pone.0101279 24983999 PMC4077732

[82] Arellano-Reynoso B, Lapaque N, Salcedo S, et al. Cyclic beta-1,2-glucan is a *Brucella* virulence factor required for intracellular survival. Nat Immunol. 2005;6(6):618–625. doi: 10.1038/ni1202 15880113

[83] Briones G, Iñón de Iannino N, Roset M, et al. *Brucella* abortus cyclic beta-1,2-glucan mutants have reduced virulence in mice and are defective in intracellular replication in HeLa cells. Infect Immun. 2001;69(7):4528–4535. doi: 10.1128/IAI.69.7.4528-4535.2001 11401996 PMC98529

[84] Sedzicki J, Ni D, Lehmann F, et al. Mechanism of cyclic beta-glucan export by ABC transporter Cgt of *Brucella*. Nat Struct Mol Biol. 2022;29(12):1170–1177. doi: 10.1038/s41594-022-00868-7 36456825

[85] Degos C, Gagnaire A, Banchereau R, et al. *Brucella* CbetaG induces a dual pro- and anti-inflammatory response leading to a transient neutrophil recruitment. Virulence. 2015;6(1):19–28. doi: 10.4161/21505594.2014.979692 25654761 PMC4603436

[86] Martirosyan A, Pérez-Gutierrez C, Banchereau R, et al. *Brucella* beta 1,2 cyclic glucan is an activator of human and mouse dendritic cells. PLOS Pathog. 2012;8(11):e1002983. doi: 10.1371/journal.ppat.1002983 23166489 PMC3499565

[87] Kambarev S, Borghesan E, Miller CN, et al. The *Brucella* abortus type IV effector BspA inhibits MARCH6-dependent ERAD to promote intracellular growth. Infect Immun. 2023;91(5):e0013023. doi: 10.1128/iai.00130-23 37129527 PMC10187129

[88] Myeni S, Child R, Ng TW, et al. *Brucella* modulates secretory trafficking via multiple type IV secretion effector proteins. PLoS Pathog. 2013;9(8):e1003556. doi: 10.1371/journal.ppat.1003556 23950720 PMC3738490

[89] Miller CN, Smith EP, Cundiff JA, et al. A *Brucella* type IV effector targets the COG tethering complex to remodel host secretory traffic and promote intracellular replication. Cell Host Microbe. 2017;22(3):317–329 e7. doi: 10.1016/j.chom.2017.07.017 28844886 PMC5599354

[90] Lin R, Li A, Li Y, et al. The *Brucella* effector protein BspF regulates apoptosis through the crotonylation of p53. Microorganisms. 2023;11(9):2322. doi: 10.3390/microorganisms11092322 37764165 PMC10534853

[91] Borghesan E, Smith EP, Myeni S, et al. A *Brucella* effector modulates the Arf6-Rab8a GTPase cascade to promote intravacuolar replication. Embo J. 2021;40(19):e107664. doi: 10.15252/embj.2021107664 34423453 PMC8488576

[92] Ma Z, Deng X, Li R, et al. Crosstalk of *Brucella* abortus nucleomodulin BspG and host DNA replication process/mitochondrial respiratory pathway promote anti-apoptosis and infection. Vet Microbiol. 2022;268:109414. doi: 10.1016/j.vetmic.2022.109414 35395545

[93] Ma Z, Li R, Hu R, et al. *Brucella* abortus BspJ is a nucleomodulin that inhibits macrophage apoptosis and promotes intracellular survival of *Brucella*. Front Microbiol. 2020;11:599205. doi: 10.3389/fmicb.2020.599205 33281799 PMC7688787

[94] Ma Z, Yu S, Cheng K, et al. Nucleomodulin BspJ as an effector promotes the colonization of *Brucella* abortus in the host. J Vet Sci. 2022;23(1):e8. doi: 10.4142/jvs.21224 34841746 PMC8799945

[95] Luizet JB, Raymond J, Lacerda TLS, et al. The *Brucella* effector BspL targets the ER-associated degradation (ERAD) pathway and delays bacterial egress from infected cells. Proc Natl Acad Sci USA. 2021;118(32). doi: 10.1073/pnas.2105324118 PMC8364137 34353909

[96] Fugier E, Salcedo SP, de Chastellier C, et al. The glyceraldehyde-3-phosphate dehydrogenase and the small GTPase Rab 2 are crucial for *Brucella* replication. PLOS Pathog. 2009;5(6):e1000487. doi: 10.1371/journal.ppat.1000487 19557163 PMC2695806

[97] de Barsy M, Jamet A, Filopon D, et al. Identification of a *Brucella* spp. secreted effector specifically interacting with human small GTPase Rab2. Cell Microbiol. 2011;13(7):1044–1058. doi: 10.1111/j.1462-5822.2011.01601.x 21501366

[98] Ke Y, Wang Y, Li W, et al. Type IV secretion system of *Brucella* spp. and its effectors. Front Cell Infect Microbiol. 2015;5:72. doi: 10.3389/fcimb.2015.00072 26528442 PMC4602199

[99] Smith EP, Cotto-Rosario A, Borghesan E, et al. Epistatic interplay between type IV secretion effectors engages the small GTPase Rab2 in the *Brucella* intracellular cycle. MBio. 2020;11(2). doi: 10.1128/mBio.03350-19 PMC7157780 32234817

[100] Barquero-Calvo E, Chaves-Olarte E, Weiss DS, et al. *Brucella* abortus uses a stealthy strategy to avoid activation of the innate immune system during the onset of infection. PLOS ONE. 2007;2(7):e631. doi: 10.1371/journal.pone.0000631 17637846 PMC1910614

[101] Sha Z, Stabel TJ, Mayfield JE. *Brucella* abortus catalase is a periplasmic protein lacking a standard signal sequence. J Bacteriol. 1994;176(23):7375–7377. doi: 10.1128/jb.176.23.7375-7377.1994 7961511 PMC197128

[102] Steele KH, Baumgartner JE, Valderas MW, et al. Comparative study of the roles of AhpC and KatE as respiratory antioxidants in *Brucella* abortus 2308. J Bacteriol. 2010;192(19):4912–4922. doi: 10.1128/JB.00231-10 20675478 PMC2944529

[103] Gee JM, Valderas MW, Kovach ME, et al. The *Brucella* abortus Cu,Zn superoxide dismutase is required for optimal resistance to oxidative killing by murine macrophages and wild-type virulence in experimentally infected mice. Infect Immun. 2005;73(5):2873–2880. doi: 10.1128/IAI.73.5.2873-2880.2005 15845493 PMC1087332

[104] Martin DW, Baumgartner JE, Gee JM, et al. Soda is a major metabolic antioxidant in *Brucella* abortus 2308 that plays a significant, but limited, role in the virulence of this strain in the mouse model. Microbiol (read). 2012;158(7):1767–1774. doi: 10.1099/mic.0.059584-0 PMC3542146 22556360

[105] Zhang G, Hu H, Yin Y, et al. *Brucella* manipulates host cell ferroptosis to facilitate its intracellular replication and egress in RAW264.7 macrophages. Antioxid (Basel). 2024;13(5):577. doi: 10.3390/antiox13050577 PMC11118192 38790682

[106] Zhai Y, Wang H, Zhang G, et al. Transcription factor OxyR regulates peroxidase levels to enhance *Brucella*’s defense against oxidative stress. Vet Microbiol. 2025;309:110673. doi: 10.1016/j.vetmic.2025.110673 40803169

[107] King KA, Caudill MT, Caswell CC. A comprehensive review of small regulatory RNAs in *Brucella* spp. Front Vet Sci. 2022;9:1026220. doi: 10.3389/fvets.2022.1026220 36532353 PMC9751625

[108] Caswell CC, Gaines JM, Ciborowski P, et al. Identification of two small regulatory RNAs linked to virulence in *Brucella* abortus 2308. Mol Microbiol. 2012;85(2):345–360. doi: 10.1111/j.1365-2958.2012.08117.x 22690807 PMC3391331

[109] Sheehan LM, Caswell CC. A 6-nucleotide regulatory motif within the AbcR small RNAs of *Brucella* abortus mediates host-pathogen interactions. MBio. 2017;8(3). doi: 10.1128/mBio.00473-17 PMC5461406 28588127

[110] Dong H, Peng X, Liu Y, et al. BASI74, a virulence-related sRNA in *Brucella* abortus. Front Microbiol. 2018;9:2173. doi: 10.3389/fmicb.2018.02173 30271397 PMC6146029

[111] Zhong Z, Xu X, Li X, et al. Large-scale identification of small noncoding RNA with strand-specific deep sequencing and characterization of a novel virulence-related sRNA in *Brucella* melitensis. Sci Rep. 2016;6(1):25123. doi: 10.1038/srep25123 27112796 PMC4845025

[112] Wang Y, Ke Y, Xu J, et al. Identification of a novel small non-coding RNA modulating the intracellular survival of *Brucella* melitensis. Front Microbiol. 2015;6:164. doi: 10.3389/fmicb.2015.00164 25852653 PMC4365724

[113] Xu D, Song J, Li G, et al. A novel small RNA Bmsr1 enhances virulence in *Brucella* melitensis M28. Vet Microbiol. 2018;223:1–8. doi: 10.1016/j.vetmic.2018.07.007 30173733

[114] King KA, Benton AH, Caudill MT, et al. Post-transcriptional control of the essential enzyme MurF by a small regulatory RNA in *Brucella* abortus. Mol Microbiol. 2024;121(1):129–141. doi: 10.1111/mmi.15207 38082493 PMC13316743

[115] Papenfort K, Bassler BL. Quorum sensing signal-response systems in Gram-negative bacteria. Nat Rev Microbiol. 2016;14(9):576–588. doi: 10.1038/nrmicro.2016.89 27510864 PMC5056591

[116] Taminiau B, Daykin M, Swift S, et al. Identification of a quorum-sensing signal molecule in the facultative intracellular pathogen *Brucella* melitensis. Infect Immun. 2002;70(6):3004–3011. doi: 10.1128/IAI.70.6.3004-3011.2002 12010991 PMC128001

[117] Delrue RM, Deschamps C, Leonard S, et al. A quorum-sensing regulator controls expression of both the type IV secretion system and the flagellar apparatus of *Brucella* melitensis. Cell Microbiol. 2005;7(8):1151–1161. doi: 10.1111/j.1462-5822.2005.00543.x 16008582

[118] Liu Y, Sun J, Peng X, et al. Deletion of the LuxR-type regulator VjbR in *Brucella* canis affects expression of type IV secretion system and bacterial virulence, and the mutant strain confers protection against *Brucella* canis challenge in mice. Microb Pathog. 2020;139:103865. doi: 10.1016/j.micpath.2019.103865 31715318

[119] Uzureau S, Lemaire J, Delaive E, et al. Global analysis of quorum sensing targets in the intracellular pathogen *Brucella* melitensis 16 M. J Proteome Res. 2010;9(6):3200–3217. doi: 10.1021/pr100068p 20387905 PMC2880877

[120] Caudill MT, Stoyanof ST, Caswell CC. Quorum sensing LuxR proteins VjbR and BabR jointly regulate *Brucella* abortus survival during infection. J Bacteriol. 2025;207(3):e0052724. doi: 10.1128/jb.00527-24 40013834 PMC11925318

[121] Borriello G, Russo V, Paradiso R, et al. Different impacts of MucR binding to the babR and virB promoters on gene expression in *Brucella* abortus 2308. Biomolecules. 2020;10(5):788. doi: 10.3390/biom10050788 32438765 PMC7277663

[122] Li Z, Wang S, Han J, et al. Deletion of *Brucella* transcriptional regulator GntR10 regulated the expression of quorum sensing system and type IV secretion system effectors, which affected the activation of NF-kappaB. J Proteomics. 2023;283–284:104938. doi: 10.1016/j.jprot.2023.104938 37230328

[123] Li Z, Wang S, Zhang H, et al. Transcriptional regulator GntR of *Brucella* abortus regulates cytotoxicity, induces the secretion of inflammatory cytokines and affects expression of the type IV secretion system and quorum sensing system in macrophages. World J Microbiol Biotechnol. 2017;33(3):60. doi: 10.1007/s11274-017-2230-9 28243986

[124] Viadas C, Rodríguez MC, Sangari FJ, et al. Transcriptome analysis of the *Brucella* abortus BvrR/BvrS two-component regulatory system. PLOS ONE. 2010;5(4):e10216. doi: 10.1371/journal.pone.0010216 20422049 PMC2858072

[125] Kleinman CL, Sycz G, Bonomi HR, et al. ChIP-seq analysis of the LuxR-type regulator VjbR reveals novel insights into the *Brucella* virulence gene expression network. Nucleic Acids Res. 2017;45(10):5757–5769. doi: 10.1093/nar/gkx165 28334833 PMC5449634

[126] Marchesini MI, Morrone Seijo SM, Guaimas FF, et al. A T4SS effector targets host cell alpha-enolase contributing to *Brucella* abortus intracellular lifestyle. Front Cell Infect Microbiol. 2016;6:153. doi: 10.3389/fcimb.2016.00153 27900285 PMC5110553

[127] Tang T, Chen G, Guo A, et al. Comparative proteomic and genomic analyses of *Brucella* abortus biofilm and planktonic cells. Mol Med Rep. 2020;21(2):731–743. doi: 10.3892/mmr.2019.10888 31974592 PMC6947884

[128] Han X, Ding C, Chen H, et al. Enzymatic and biological characteristics of enolase in *Brucella* abortus A19. Mol Biol Rep. 2012;39(3):2705–2711. doi: 10.1007/s11033-011-1025-6 21674187

[129] Abbady AQ, Al-Daoude A, Al-Mariri A, et al. Chaperonin GroEL a *Brucella* immunodominant antigen identified using nanobody and MALDI-TOF-MS technologies. Vet Immunol Immunopathol. 2012;146(3–4):254–263. doi: 10.1016/j.vetimm.2012.01.015 22472910

[130] Meylan E, Tschopp J, Karin M. Intracellular pattern recognition receptors in the host response. Nature. 2006;442(7098):39–44. doi: 10.1038/nature04946 16823444

[131] Gomes MT, Campos PC, de Almeida LA, et al. The role of innate immune signals in immunity to *Brucella* abortus. Front Cell Infect Microbiol. 2012;2:130. doi: 10.3389/fcimb.2012.00130 23112959 PMC3480720

[132] Wei Z, Zhang S, Wang X, et al. Beyond survival to domination: brucella’s multilayered strategies for evading host immune responses. Front Microbiol. 2025;16:1608617. doi: 10.3389/fmicb.2025.1608617 40606156 PMC12213604

[133] Akira S, Uematsu S, Takeuchi O. Pathogen recognition and innate immunity. Cell. 2006;124(4):783–801. doi: 10.1016/j.cell.2006.02.015 16497588

[134] Kawai T, Akira S. The role of pattern-recognition receptors in innate immunity: update on toll-like receptors. Nat Immunol. 2010;11(5):373–384. doi: 10.1038/ni.1863 20404851

[135] Cardoso PG, Macedo GC, Azevedo V, et al. *Brucella* spp noncanonical LPS: structure, biosynthesis, and interaction with host immune system. Microb Cell Fact. 2006;5(1):13. doi: 10.1186/1475-2859-5-13 16556309 PMC1435926

[136] Lapaque N, Takeuchi O, Corrales F, et al. Differential inductions of TNF-alpha and IGTP, IIGP by structurally diverse classic and non-classic lipopolysaccharides. Cell Microbiol. 2006;8(3):401–413. doi: 10.1111/j.1462-5822.2005.00629.x 16469053

[137] Byndloss MX, Tsolis RM. *Brucella* spp. virulence factors and immunity. Annu Rev Anim Biosci. 2016;4(1):111–127. doi: 10.1146/annurev-animal-021815-111326 26734887

[138] Hagar JA, Powell DA, Aachoui Y, et al. Cytoplasmic LPS activates caspase-11: implications in TLR4-independent endotoxic shock. Science. 2013;341(6151):1250–1253. doi: 10.1126/science.1240988 24031018 PMC3931427

[139] Kayagaki N, Wong MT, Stowe IB, et al. Noncanonical inflammasome activation by intracellular LPS independent of TLR4. Science. 2013;341(6151):1246–1249. doi: 10.1126/science.1240248 23887873

[140] Cerqueira DM, Gomes MTR, Silva ALN, et al. Guanylate-binding protein 5 licenses caspase-11 for gasdermin-D mediated host resistance to *Brucella* abortus infection. PLOS Pathog. 2018;14(12):e1007519. doi: 10.1371/journal.ppat.1007519 30589883 PMC6326519

[141] Jakka P, Namani S, Murugan S, et al. The *Brucella* effector protein TcpB induces degradation of inflammatory caspases and thereby subverts non-canonical inflammasome activation in macrophages. J Biol Chem. 2017;292(50):20613–20627. doi: 10.1074/jbc.M117.815878 29061850 PMC5733597

[142] Smith KD, Ozinsky A. Toll-like receptor-5 and the innate immune response to bacterial flagellin. Curr Top Microbiol Immunol. 2002;270:93–108. 12467246 10.1007/978-3-642-59430-4_6

[143] Eaves-Pyles T, Murthy K, Liaudet L, et al. Flagellin, a novel mediator of Salmonella-induced epithelial activation and systemic inflammation: i kappa B alpha degradation, induction of nitric oxide synthase, induction of proinflammatory mediators, and cardiovascular dysfunction. J Immunol. 2001;166(2):1248–1260. doi: 10.4049/jimmunol.166.2.1248 11145708

[144] Andersen-Nissen E, Smith KD, Strobe KL, et al. Evasion of toll-like receptor 5 by flagellated bacteria. Proc Natl Acad Sci USA. 2005;102(26):9247–9252. doi: 10.1073/pnas.0502040102 15956202 PMC1166605

[145] Terwagne M, Ferooz J, Rolán HG, et al. Innate immune recognition of flagellin limits systemic persistence of *Brucella*. Cell Microbiol. 2013;15(6):942–960. doi: 10.1111/cmi.12088 23227931 PMC4026035

[146] Li W, Ke Y, Wang Y, et al. *Brucella* TIR-like protein TcpB/Btp1 specifically targets the host adaptor protein MAL/TIRAP to promote infection. Biochem Biophys Res Commun. 2016;477(3):509–514. doi: 10.1016/j.bbrc.2016.06.064 27311859

[147] Zhang J, Guo F, Huang X, et al. A novel Omp25-binding peptide screened by phage display can inhibit *Brucella* abortus 2308 infection in vitro and in vivo. J Med Microbiol. 2014;63(6):780–787. doi: 10.1099/jmm.0.069559-0 24722798 PMC4030397

[148] Luo X, Zhang X, Wu X, et al. *Brucella* downregulates tumor necrosis factor-alpha to promote intracellular survival via Omp25 regulation of different microRNAs in porcine and murine macrophages. Front Immunol. 2017;8:2013. doi: 10.3389/fimmu.2017.02013 29387067 PMC5776175

[149] Jubier-Maurin V, Boigegrain R-A, Cloeckaert A, et al. Major outer membrane protein Omp25 of *Brucella* suis is involved in inhibition of tumor necrosis factor alpha production during infection of human macrophages. Infect Immun. 2001;69(8):4823–4830. doi: 10.1128/IAI.69.8.4823-4830.2001 11447156 PMC98570

[150] Kutlu M, Ergin Ç, Sen-Türk N, et al. Acute *Brucella* melitensis M16 infection model in mice treated with tumor necrosis factor-alpha inhibitors. J Infect Dev Ctries. 2015;9(2):141–148. doi: 10.3855/jidc.5155 25699488

[151] Cui B, Liu W, Wang X, et al. *Brucella* Omp25 upregulates miR-155, miR-21-5p, and miR-23b to inhibit interleukin-12 production via modulation of programmed death-1 signaling in human monocyte/macrophages. Front Immunol. 2017;8:708. doi: 10.3389/fimmu.2017.00708 28694807 PMC5483987

[152] Kanayama A, Seth RB, Sun L, et al. Tab2 and Tab3 activate the NF-kappaB pathway through binding to polyubiquitin chains. Mol Cell. 2004;15(4):535–548. doi: 10.1016/j.molcel.2004.08.008 15327770

[153] Degos C, Hysenaj L, Gonzalez‐Espinoza G, et al. Omp25-dependent engagement of SLAMF1 by *Brucella* abortus in dendritic cells limits acute inflammation and favours bacterial persistence in vivo. Cell Microbiol. 2020;22(4):e13164. doi: 10.1111/cmi.13164 31953913

[154] Murugan S, Nandi BR, Mazumdar V, et al. Outer membrane protein 25 of *Brucella* suppresses TLR-mediated expression of proinflammatory cytokines through degradation of TLRs and adaptor proteins. J Biol Chem. 2023;299(11):105309. doi: 10.1016/j.jbc.2023.105309 37778729 PMC10641269

[155] Li R, Liu W, Yin X, et al. *Brucella* spp. Omp25 promotes proteasome-mediated cGAS degradation to attenuate IFN-beta production. Front Microbiol. 2021;12:702881. doi: 10.3389/fmicb.2021.702881 34394047 PMC8358459

[156] Verdiguel-Fernandez L, Oropeza-Navarro R, Ortiz-Rico A, et al. *Brucella* melitensis omp31 mutant is attenuated and confers protection against virulent *Brucella* melitensis challenge in BALB/c mice. J Microbiol Biotechnol. 2020;30(4):497–504. doi: 10.4014/jmb.1908.08056 31986561 PMC9728373

[157] Vizcaino N, Verger J-M, Grayon M, et al. Dna polymorphism at the omp-31 locus of *Brucella* spp.: evidence for a large deletion in *Brucella* abortus, and other species-specific markers. Microbiol (read). 1997;143(9):2913–2921. doi: 10.1099/00221287-143-9-2913 9308175

[158] Wang Z, Wang G, Wang Y, et al. Omp31 of *Brucella* inhibits NF-kappaB p65 signaling pathway by inducing autophagy in BV-2 microglia. Neurochem Res. 2021;46(12):3264–3272. doi: 10.1007/s11064-021-03429-4 34536195

[159] Zhang K, Wang H, Guo F, et al. Omp31 of *Brucella* melitensis 16M impairs the apoptosis of macrophages triggered by TNF-alpha. Exp Ther Med. 2016;12(4):2783–2789. doi: 10.3892/etm.2016.3655 27698784 PMC5038375

[160] Khan M, Harms JS, Liu Y, et al. *Brucella* suppress sting expression via miR-24 to enhance infection. PLOS Pathog. 2020;16(10):e1009020. doi: 10.1371/journal.ppat.1009020 33108406 PMC7647118

[161] Hebert DN, Molinari M. In and out of the ER: protein folding, quality control, degradation, and related human diseases. Physiol Rev. 2007;87(4):1377–1408. doi: 10.1152/physrev.00050.2006 17928587

[162] Hetz C. The unfolded protein response: controlling cell fate decisions under ER stress and beyond. Nat Rev Mol Cell Biol. 2012;13(2):89–102. doi: 10.1038/nrm3270 22251901

[163] Walter P, Ron D. The unfolded protein response: from stress pathway to homeostatic regulation. Science. 2011;334(6059):1081–1086. doi: 10.1126/science.1209038 22116877

[164] Di Conza G, Ho P-C, Cubillos-Ruiz JR, et al. Control of immune cell function by the unfolded protein response. Nat Rev Immunol. 2023;23(9):546–562. doi: 10.1038/s41577-023-00838-0 36755160

[165] Celli J, Tsolis RM. Bacteria, the endoplasmic reticulum and the unfolded protein response: friends or foes? Nat Rev Microbiol. 2015;13(2):71–82. doi: 10.1038/nrmicro3393 25534809 PMC4447104

[166] Qin QM, Pei J, Ancona V, et al. Rnai screen of endoplasmic reticulum-associated host factors reveals a role for IRE1alpha in supporting *Brucella* replication. PLOS Pathog. 2008;4(7):e1000110. doi: 10.1371/journal.ppat.1000110 18654626 PMC2453327

[167] Guimaraes ES, Gomes MTR, Campos PC, et al. *Brucella* abortus cyclic dinucleotides trigger STING-dependent unfolded protein response that favors bacterial replication. J Immunol. 2019;202(9):2671–2681. doi: 10.4049/jimmunol.1801233 30894428 PMC6478548

[168] Bronner DN, Abuaita B, Chen X, et al. Endoplasmic reticulum stress activates the inflammasome via NLRP3- and caspase-2-driven mitochondrial damage. Immunity. 2015;43(3):451–462. doi: 10.1016/j.immuni.2015.08.008 26341399 PMC4582788

[169] Li C, Wang J, Sun W, et al. The *Brucella* effector BspI suppresses inflammation via inhibition of IRE1 kinase activity during *Brucella* infection. J Immunol. 2022;209(3):488–497. doi: 10.4049/jimmunol.2200001 35840160

[170] Taguchi Y, Imaoka K, Kataoka M, et al. Yip1A, a novel host factor for the activation of the IRE1 pathway of the unfolded protein response during *Brucella* infection. PLOS Pathog. 2015;11(3):e1004747. doi: 10.1371/journal.ppat.1004747 25742138 PMC4350842

[171] Wells KM, He K, Pandey A, et al. *Brucella* activates the host RIDD pathway to subvert BLOS1-directed immune defense. Elife. 2022;11. doi: 10.7554/eLife.73625 PMC9119680 35587649

[172] Pandey A, Lin F, Cabello AL, et al. Activation of host IRE1alpha-dependent signaling axis contributes the intracellular parasitism of *Brucella* melitensis. Front Cell Infect Microbiol. 2018;8:103. doi: 10.3389/fcimb.2018.00103 29732320 PMC5919948

[173] Gomes MTR, Guimarães ES, Marinho FV, et al. Sting regulates metabolic reprogramming in macrophages via HIF-1alpha during *Brucella* infection. PLOS Pathog. 2021;17(5):e1009597. doi: 10.1371/journal.ppat.1009597 33989349 PMC8153530

[174] Guimaraes ES, Gomes MTR, Sanches RCO, et al. The endoplasmic reticulum stress sensor IRE1alpha modulates macrophage metabolic function during *Brucella* abortus infection. Front Immunol. 2022;13:1063221. doi: 10.3389/fimmu.2022.1063221 36660548 PMC9842658

[175] Guimaraes ES, Gomes MTR, Moraes-Vieira PM, et al. *Brucella* abortus induces dynamin-related protein 1 (DRP1)-dependent mitochondrial fission in infected macrophages via stress sensor IRE1alpha altering metabolic function. J Immunol. 2025;214(10):2688–2699. doi: 10.1093/jimmun/vkaf198 40811739 PMC12576116

[176] Qin Y, Zhou G, Jiao F, et al. *Brucella* mediates autophagy, inflammation, and apoptosis to escape host killing. Front Cell Infect Microbiol. 2024;14:1408407. doi: 10.3389/fcimb.2024.1408407 39507949 PMC11537862

[177] Ahmed W, Zheng K, Liu ZF. Establishment of chronic infection: brucella’s stealth strategy. Front Cell Infect Microbiol. 2016;6:30. 27014640 10.3389/fcimb.2016.00030 PMC4791395

[178] Guo F, Zhang H, Chen C, et al. Autophagy favors *Brucella* melitensis survival in infected macrophages. Cell Mol Biol Lett. 2012;17(2):249–257. doi: 10.2478/s11658-012-0009-4 22367856 PMC6275789

[179] Dong B, Li F, Wang J, et al. Effect of ubiquitin-proteasome system and autophagy-lysosome pathway on intracellular replication of *Brucella*.suis. Vet Microbiol. 2023;280:109699. doi: 10.1016/j.vetmic.2023.109699 36812863

[180] Hamer I, Goffin E, De Bolle X, et al. Replication of *Brucella* abortus and *Brucella* melitensis in fibroblasts does not require Atg5-dependent macroautophagy. BMC Microbiol. 2014;14(1):223. doi: 10.1186/s12866-014-0223-5 25179110 PMC4159544

[181] Arriola Benitez PC, Pesce Viglietti AI, Herrmann CK, et al. *Brucella* abortus promotes a fibrotic phenotype in hepatic stellate cells, with concomitant activation of the autophagy pathway. Infect Immun. 2018;86(1). doi: 10.1128/IAI.00522-17 PMC5736806 28993461

[182] Louche A, Blanco A, Lacerda TLS, et al. *Brucella* effectors NyxA and NyxB target SENP3 to modulate the subcellular localisation of nucleolar proteins. Nat Commun. 2023;14(1):102. doi: 10.1038/s41467-022-35763-8 36609656 PMC9823007

[183] Jiao H, Luo Y, Zhou Z, et al. Integrative bioinformatics identification of the autophagic pathway-associated miRNA-mRNA networks in RAW264.7 macrophage cells infected with ∆Omp25 *Brucella* melitensis. Inflammation. 2020;43(2):532–539. doi: 10.1007/s10753-019-01135-6 31807961

[184] Verbeke J, De Bolle X, Arnould T. When mitophagy dictates the outcome of cellular infection: the case of *Brucella* abortus. Autophagy. 2023;19(11):3022–3023. doi: 10.1080/15548627.2023.2246354 37589593 PMC10549184

[185] Hop HT, Huy TXN, Lee HJ, et al. Intracellular growth of *Brucella* is mediated by dps-dependent activation of ferritinophagy. EMBO Rep. 2023;24(9):e55376. doi: 10.15252/embr.202255376 37503678 PMC10481649

[186] Xu H, Lu J, Huang F, et al. A genome-wide CRISPR screen identified host genes essential for intracellular *Brucella* survival. Microbiol Spectr. 2024;12(4):e0338323. doi: 10.1128/spectrum.03383-23 38376367 PMC10986529

[187] Jung KI, McKenna S, Vijayamahantesh V, et al. Protective versus pathogenic type I interferon responses during virus infections. Viruses. 2023;15(9):1916. doi: 10.3390/v15091916 37766322 PMC10538102

[188] Oliveira SC, de Oliveira FS, Macedo GC, et al. The role of innate immune receptors in the control of *Brucella* abortus infection: toll-like receptors and beyond. Microbes Infect. 2008;10(9):1005–1009. doi: 10.1016/j.micinf.2008.07.005 18664388

[189] Brierley MM, Fish EN. Review: iFN-alpha/beta receptor interactions to biologic outcomes: understanding the circuitry. J Interferon Cytokine Res. 2002;22(8):835–845. 12396722 10.1089/107999002760274845

[190] Shuai K, Liu B. Regulation of JAK-STAT signalling in the immune system. Nat Rev Immunol. 2003;3(11):900–911. doi: 10.1038/nri1226 14668806

[191] Trinchieri G. Type I interferon: friend or foe? J Exp Med. 2010;207(10):2053–2063. doi: 10.1084/jem.20101664 20837696 PMC2947062

[192] Qiu M, Li J, Wu W, et al. The dual role of type I interferons in bacterial infections: from immune defense to pathogenesis. MBio. 2025;16(7):e0148125. doi: 10.1128/mbio.01481-25 40525851 PMC12239577

[193] Rothfuchs AG, Trumstedt C, Wigzell H, et al. Intracellular bacterial infection-induced IFN-gamma is critically but not solely dependent on toll-like receptor 4-myeloid differentiation factor 88-IFN-alpha beta-STAT1 signaling. J Immunol. 2004;172(10):6345–6353. doi: 10.4049/jimmunol.172.10.6345 15128825

[194] Archer KA, Durack J, Portnoy DA. Sting-dependent type I IFN production inhibits cell-mediated immunity to Listeria monocytogenes. PLoS Pathog. 2014;10(1):e1003861. doi: 10.1371/journal.ppat.1003861 24391507 PMC3879373

[195] Snyder DT, Hedges JF, Jutila MA. Getting “inside” type I IFNs: type I IFNs in intracellular bacterial infections. J Immunol Res. 2017;2017:9361802. 28529959 10.1155/2017/9361802 PMC5424489

[196] Kotov DI, Lee OV, Fattinger SA, et al. Early cellular mechanisms of type I interferon-driven susceptibility to tuberculosis. Cell. 2023;186(25):5536–5553 e22. doi: 10.1016/j.cell.2023.11.002 38029747 PMC10757650

[197] de Almeida LA, Carvalho NB, Oliveira FS, et al. MyD88 and STING signaling pathways are required for IRF3-mediated IFN-beta induction in response to *Brucella* abortus infection. PLOS ONE. 2011;6(8):e23135. doi: 10.1371/journal.pone.0023135 21829705 PMC3149075

[198] Moreira-Teixeira L, Mayer-Barber K, Sher A, et al. Type I interferons in tuberculosis: foe and occasionally friend. J Exp Med. 2018;215(5):1273–1285. doi: 10.1084/jem.20180325 29666166 PMC5940272

[199] Ji DX, Witt KC, Kotov DI, et al. Role of the transcriptional regulator SP140 in resistance to bacterial infections via repression of type I interferons. Elife. 2021;10. doi: 10.7554/eLife.67290 PMC8248984 34151776

[200] Huang LY, Ishii KJ, Akira S, et al. Th1-like cytokine induction by heat-killed *Brucella* abortus is dependent on triggering of TLR9. J Immunol. 2005;175(6):3964–3970. doi: 10.4049/jimmunol.175.6.3964 16148144

[201] Gomes MTR, Guimaraes ES, Oliveira SC. ZBP1 senses *Brucella* abortus DNA triggering type I interferon signaling pathway and unfolded protein response activation. Front Immunol. 2024;15:1511949. 39850894 10.3389/fimmu.2024.1511949 PMC11754416

[202] Auerbuch V, Brockstedt DG, Meyer-Morse N, et al. Mice lacking the type I interferon receptor are resistant to Listeria monocytogenes. J Exp Med. 2004;200(4):527–533. doi: 10.1084/jem.20040976 15302899 PMC2211930

[203] O’Connell RM, Saha SK, Vaidya SA, et al. Type I interferon production enhances susceptibility to Listeria monocytogenes infection. J Exp Med. 2004;200(4):437–445. doi: 10.1084/jem.20040712 15302901 PMC2211937

[204] Escoll P, Buchrieser C. Metabolic reprogramming of host cells upon bacterial infection: why shift to a Warburg-like metabolism? FEBS J. 2018;285(12):2146–2160. doi: 10.1111/febs.14446 29603622

[205] Pearce EL, Pearce EJ. Metabolic pathways in immune cell activation and quiescence. Immunity. 2013;38(4):633–643. doi: 10.1016/j.immuni.2013.04.005 23601682 PMC3654249

[206] Amo-Aparicio J, Dinarello CA, Lopez-Vales R. Metabolic reprogramming of the inflammatory response in the nervous system: the crossover between inflammation and metabolism. Neural Regen Res. 2024;19(10):2189–2201. doi: 10.4103/1673-5374.391330 38488552 PMC11034585

[207] Chen S, Saeed AFUH, Liu Q, et al. Macrophages in immunoregulation and therapeutics. Signal Transduct Target Ther. 2023;8(1):207. doi: 10.1038/s41392-023-01452-1 37211559 PMC10200802

[208] Hu H, Tian M, Li P, et al. *Brucella* infection regulates thioredoxin-interacting protein expression to facilitate intracellular survival by reducing the production of nitric oxide and reactive oxygen species. J Immunol. 2020;204(3):632–643. doi: 10.4049/jimmunol.1801550 31852753

[209] Gomes MT, Campos PC, Pereira GDS, et al. Tlr9 is required for MAPK/NF-kappaB activation but does not cooperate with TLR2 or TLR6 to induce host resistance to *Brucella* abortus. J Leukoc Biol. 2016;99(5):771–780. doi: 10.1189/jlb.4A0815-346R 26578650 PMC6011818

[210] Zhao T, Zhang Z, Li Y, et al. *Brucella* abortus modulates macrophage polarization and inflammatory response by targeting glutaminases through the NF-kappaB signaling pathway. Front Immunol. 2023;14:1180837. doi: 10.3389/fimmu.2023.1180837 37325614 PMC10266586

[211] Xavier MN, Winter M, Spees A, et al. Ppargamma-mediated increase in glucose availability sustains chronic *Brucella* abortus infection in alternatively activated macrophages. Cell Host Microbe. 2013;14(2):159–170. doi: 10.1016/j.chom.2013.07.009 23954155 PMC3777723

[212] Kerrinnes T, Winter MG, Young BM, et al. Utilization of host polyamines in alternatively activated macrophages promotes chronic infection by *Brucella* abortus. Infect Immun. 2018;86(3). doi: 10.1128/IAI.00458-17 PMC5820950 29203548

[213] Czyz DM, Willett JW, Crosson S. *Brucella* abortus induces a Warburg shift in host metabolism that is linked to enhanced intracellular survival of the pathogen. J Bacteriol. 2017;199(15). doi: 10.1128/JB.00227-17 PMC5512224 28559292

[214] Cao S, Guo J, Zhu D, et al. *Brucella* induced upregulation of no promote macrophages glycolysis through the NF-kappaB/G6PD pathway. Int Immunopharmacol. 2024;142(Pt A):113038. doi: 10.1016/j.intimp.2024.113038 39276450

[215] Lacey CA, Ponzilacqua-Silva B, Chambers CA, et al. MyD88-dependent glucose restriction and itaconate production control *Brucella* infection. Infect Immun. 2021;89(10):e0015621. doi: 10.1128/IAI.00156-21 34125603 PMC8445166

[216] Homberger C, Barquist L, Vogel J. Ushering in a new era of single-cell transcriptomics in bacteria. Microlife. 2022;3:uqac020. doi: 10.1093/femsml/uqac020 37223351 PMC10117829

[217] Hop HT, Arayan LT, Reyes AWB, et al. Simultaneous RNA-seq based transcriptional profiling of intracellular *Brucella* abortus and B. abortus-infected murine macrophages. Microb Pathog. 2017;113:57–67. doi: 10.1016/j.micpath.2017.10.029 29054743

[218] Wang Y, Yang S, Han B, et al. Single-cell landscape revealed immune characteristics associated with disease phases in brucellosis patients. Imeta. 2024;3(4):e226. doi: 10.1002/imt2.226 39135683 PMC11316929

[219] Mortier T, Wieme AD, Vandamme P, et al. Bacterial species identification using MALDI-TOF mass spectrometry and machine learning techniques: a large-scale benchmarking study. Comput Struct Biotechnol J. 2021;19:6157–6168. doi: 10.1016/j.csbj.2021.11.004 34938408 PMC8649224

[220] Dematheis F, Walter MC, Lang D, et al. Machine learning algorithms for classification of MALDI-TOF ms spectra from phylogenetically closely related species *Brucella* melitensis, *Brucella* abortus and *Brucella* suis. Microorganisms. 2022;10(8):1658. doi: 10.3390/microorganisms10081658 36014076 PMC9416640

[221] Hashemzadeh P, Nezhad SA, Khoshkhabar H. Immunoinformatics analysis of *Brucella* melitensis to approach a suitable vaccine against brucellosis. J Genet Eng Biotechnol. 2023;21(1):152. doi: 10.1186/s43141-023-00614-6 38019359 PMC10686926

[222] Gharazi H, Doosti A, Abdizadeh R. Brucellosis novel multi-epitope vaccine design based on in silico analysis focusing on *Brucella* abortus. BMC Immunol. 2025;26(1):46. doi: 10.1186/s12865-025-00728-1 40610899 PMC12232191

[223] Wu A, Wang Y, Ali A, et al. Design of a multi-epitope vaccine against brucellosis fused to IgG-Fc by an immunoinformatics approach. Front Vet Sci. 2023;10:1238634. doi: 10.3389/fvets.2023.1238634 37937155 PMC10625910

[224] He Y. Analyses of *Brucella* pathogenesis, host immunity, and vaccine targets using systems biology and bioinformatics. Front Cell Infect Microbiol. 2012;2:2. doi: 10.3389/fcimb.2012.00002 22919594 PMC3417401

[225] Ong E, He Y. Vaccine design by reverse vaccinology and machine learning. Methods Mol Biol. 2022;2414:1–16. 34784028 10.1007/978-1-0716-1900-1_1 PMC12046528

[226] Ong E, Wang H, Wong MU, et al. Vaxign-ML: supervised machine learning reverse vaccinology model for improved prediction of bacterial protective antigens. Bioinformatics. 2020;36(10):3185–3191. doi: 10.1093/bioinformatics/btaa119 32096826 PMC7214037

[227] Blasco JM, Moreno E, Muñoz PM, et al. A review of three decades of use of the cattle brucellosis rough vaccine *Brucella* abortus RB51: myths and facts. BMC Vet Res. 2023;19(1):211. doi: 10.1186/s12917-023-03773-3 37853407 PMC10583465

[228] Heidary M, Dashtbin S, Ghanavati R, et al. Evaluation of brucellosis vaccines: a comprehensive review. Front Vet Sci. 2022;9:925773. doi: 10.3389/fvets.2022.925773 35923818 PMC9339783

[229] Shi J, Zhu Y, Yin Z, et al. In silico designed novel multi-epitope mRNA vaccines against *Brucella* by targeting extracellular protein BtuB and LptD. Sci Rep. 2024;14(1):7278. doi: 10.1038/s41598-024-57793-6 38538674 PMC10973489

[230] Asadinezhad M, Pakzad I, Asadollahi P, et al. Proteomics exploration of *Brucella* melitensis to design an innovative multi-epitope mRNA vaccine. Bioinform Biol Insights. 2024;18:11779322241272404. doi: 10.1177/11779322241272404 39220468 PMC11365029

[231] Zhu Y, Shi J, Wang Q, et al. Novel dual-pathogen multi-epitope mRNA vaccine development for *Brucella* melitensis and Mycobacterium tuberculosis in silico approach. PLOS ONE. 2024;19(10):e0309560. doi: 10.1371/journal.pone.0309560 39466745 PMC11515988

